# Intra-tumor heterogeneity, turnover rate and karyotype space shape susceptibility to missegregation-induced extinction

**DOI:** 10.1371/journal.pcbi.1010815

**Published:** 2023-01-23

**Authors:** Gregory J. Kimmel, Richard J. Beck, Xiaoqing Yu, Thomas Veith, Samuel Bakhoum, Philipp M. Altrock, Noemi Andor

**Affiliations:** 1 H. Lee Moffitt Cancer Center & Research Institute, Integrated Mathematical Oncology, Tampa, Florida; 2 H. Lee Moffitt Cancer Center & Research Institute, Biostatistics & Bioinformatics, Tampa, Florida; 3 Memorial Sloan Kettering Cancer Center, Human Oncology & Pathogenesis Program, New York City, New York; 4 Max Planck Institute for Evolutionary Biology, Department for Evolutionary Theory, Plön, Germany; University of Illinois at Urbana-Champaign, UNITED STATES

## Abstract

The phenotypic efficacy of somatic copy number alterations (SCNAs) stems from their incidence per base pair of the genome, which is orders of magnitudes greater than that of point mutations. One mitotic event stands out in its potential to significantly change a cell’s SCNA burden–a chromosome missegregation. A stochastic model of chromosome mis-segregations has been previously developed to describe the evolution of SCNAs of a single chromosome type. Building upon this work, we derive a general deterministic framework for modeling missegregations of multiple chromosome types. The framework offers flexibility to model intra-tumor heterogeneity in the SCNAs of all chromosomes, as well as in missegregation- and turnover rates. The model can be used to test how selection acts upon coexisting karyotypes over hundreds of generations. We use the model to calculate missegregation-induced population extinction (MIE) curves, that separate viable from non-viable populations as a function of their turnover- and missegregation rates. Turnover- and missegregation rates estimated from scRNA-seq data are then compared to theoretical predictions. We find convergence of theoretical and empirical results in both the location of MIE curves and the necessary conditions for MIE. When a dependency of missegregation rate on karyotype is introduced, karyotypes associated with low missegregation rates act as a stabilizing refuge, rendering MIE impossible unless turnover rates are exceedingly high. Intra-tumor heterogeneity, including heterogeneity in missegregation rates, increases as tumors progress, rendering MIE unlikely.

## Author summary

Chromosome missegregations are common. They occur in 1.2–2.3% per mitosis in normal cells and in cancer cells their rate is between one and two orders of magnitudes higher. When a cell missegregates a chromosome while dividing, the chance is high that its two daughter cells will behave drastically different from each other and from their parental cell. Chromosome missegregations are therefore one of the most powerful forces of phenotypic diversity. We developed a mathematical model of chromosome missegregations that allows for this cell-to-cell diversity to be accounted for. The model serves to help understand how selection acts upon cells with versatile chromosome contents, as a tool for genotype-to-phenotype mapping in various microenvironments. As a first application example we used the model to address whether there exists an upper limit on missegregation rate, beyond which cancer populations collapse. The model revealed that the upper limit of missegregation rate is a function of the tumor’s turnover rate (i.e. how fast the tumor renews itself). In heterogenous populations however, cells with low missegregation rates protect the population from collapse. Intra-tumor heterogeneity, including heterogeneity in missegregation rates, increases as tumors progress, rendering missegregation-induced extinction unlikely.

## 1 Introduction

Aneuploidy, defined as a chromosome number that is not the exact multiple of the haploid karyotype, is common across several cancers, including non-small-cell lung, breast, colorectal, prostate cancer and glioblastoma [1–5]. The main driver of aneuploidy is chromosomal instability (CIN). CIN-induced genomic changes can be subdivided into two categories: the whole gain or loss of a chromosome (numerical CIN) or changes within localized regions of a chromosome (structural CIN).

Thompson and Compton used live cell imaging to evaluate the fidelity of chromosome segregation, finding missegregation rates ranging from 0.025–1% per chromosome per mitosis [6]. We distinguish between unpredictable and predictable factors governing a cell’s risk to missegregate. Unpredictable events include DNA double-strand breaks (DSBs). Their location in the DNA appears to be random, yet has been shown to influence the likelihood of mitotic delay and subsequent missegregation events [7–10]. This delay allows for DNA damage response (DDR) during mitosis and thus protects the genome from structural damage, but at the expense of increasing risk for numerical instability [11]. Predictable factors that increase the incidence of missegregations include high ploidy [12] and suboptimal kinetochore-microtubule attachment stability. Tetraploid cells are more likely to fail to cluster centrosomes into two poles, leading to multipolar division. While multipolar divisions are likely lethal, multipolar mitosis can also cause the poles to coalesce leading to a pseudobipolar division and chromosome missegregations ([13]). Kinetochore-microtubule attachment stability must fall within a narrow permissible window to allow for faithful chromosome segregation [11, 14–16] and is influenced by both *cell-intrinsic* [11] and *extrinsic* factors. An example of extrinsic factors are Vinca alkaloids (e.g. vincristine, vinblastine),—a class of cytotoxic drugs which act directly upon the microtubule network [17], causing increased missegregation rates [18]. But even cytotoxic drugs not directly targeting the microtubule network have been shown to significantly impede segregation fidelity, through aforementioned stimulation of DDR and mitotic delay [19]. Drugs targeting the DDR are likely to induce numerical instability [19], suggesting that DNA-damaging therapies impart part of their cytotoxicity by interfering with chromosome segregation fidelity [20]. Changes of the tumor microenvironment such as glucose deprivation, hypoxia and acidification have also been linked to CIN [21, 22].

Anueploidy and CIN are often coupled and can create a positive-feedback loop in which further structural or whole chromosome aberrations accumulate over time [23, 24]. But normal cells do not tolerate missegregations—aneuploid daughter cells are immediately cleared from the cell pool through apoptosis during G1 following a missegregation [25, 26]. The sudden genome-dosage imbalance caused by a missegregation event induces p53-mediated cellular senescence in the subsequent G1 phase [12, 25–27]. While cancer cells evolve to more proficiently avert missegregation-induced cell death, missegregations still activate p53 in the G1 phase, even among cancer cells [27], albeit less reliably [28]. High levels of CIN have been observed to be tumor suppressive in breast [29], ovarian, gastric, and non-small cell lung cancer [30]. The above suggests that a non-monotonic relationship between cell fitness and CIN likely exists, with a threshold of a critical level of CIN (which may be cancer type specific). A possible therapeutic avenue to target and exploit the degree of CIN in patients is therefore guided by the premise that a Goldilocks window exists for cancer to thrive.

Gusev et al. [31] modeled the evolution of cell karyotypes via chromosome mis-segregations as a random branching walk. Using this model, the authors estimated the fraction of clones surviving as a function of mis-segregation rate and approximated a theoretical limit for mis-segregation rate for a diploid population to survive without a complete loss of any chromosome type. In a follow-up publication, the same authors used a semianalytical approach to analyze the asymptotic behavior of this model, simulating evolution of the copy number of just a single chromosome type [32]. They compared various mechanisms of chromosome mis-segregations with respect to their ability to generate a stable distribution of chromosome numbers. Elizalde et. al. explored the phenotypic impact of CIN using a Markov-chain model and confirmed the existence of optimal chromosome missegregation rates [33]. The authors assumed that cells were not viable if they contained nullisomy (the loss of all copies of a chromosome), paired with a corresponding upper limit of eight copies. These assumptions were justified through a sensitivity analysis [34]. The main conclusion of the paper established that missegregation rates drove heterogeneity more than the age of the tumor. Under what circumstances missegregations lead to tumor extinction however remains unclear. Here we derive necessary conditions that drive a tumor population to nonviable karyotypes, typically through either nullisomy or an upper limit on the number of sustainable copies per chromosome (e.g. eight [33]). We further refer to these conditions as missegregation-induced extinction (MIE).

The remainder of this manuscript is structured as follows. We first motivate the existence of MIE with a phenomenological equation of ploidy movement that takes the form of a diffusion-reaction equation. A heuristic argument comparing the time scales of net growth and missegregation will imply the existence of turnover and missegregation rates that allow for MIE. We then develop our mathematical framework–a general coupled compartment model of chromosome mis-segregations. This model is simplified to a version more amendable to theoretical analysis, while still retaining the qualitative behavior and form. Theoretical results are derived and presented on the existence of MIE and when it can be evaded. Next, we derive turnover and missegregation rates from a PAN-cancer scRNA-seq dataset from 14 tumors and quantify their relationship to ploidy and to the copy number of individual chromosomes. Finally, we use the scRNA-seq derived measurements to predict which tumors are most sensitive to MIE.

## 2 Results

### 2.1 Cell turnover rates and susceptibility to MIE

Consider a simple birth-death process on ploidy space, where, for the moment, we are interested only in the total amount of DNA content of a cell. If only missegregations facilitate the movement in DNA content during mitosis, one can crudely approximate the total population *n*(*p*) as a function of DNA content *p* by the following partial differential equation (PDE):

$$\begin{array}{c} \frac{\partial n}{\partial t}=\left(\lambda -\mu \right)n+\beta \lambda \frac{\partial^{2}n}{\partial p^{2}}, \end{array}$$ (1)

where λ, *μ* are the birth, death rates, respectively and *β* is the missegregation rate. For boundary conditions, we make the assumption that there exists *p* ∈ (*p*_min_, *p*_max_), such that for DNA content outside this range, the population cannot survive. We also assume that *r* = λ − *μ* > 0, that is, in the absence of missegregation, this population is favored to grow.

We now appeal to a heuristic argument of time scales. Let *T*_*p*_ be the timescale on which missegregation events happen, which, in Fickian diffusion is proportional to: $T_{p}∼L_{p}^{2}$, where *L*_*p*_ is the characteristic amount of DNA content shifted during a missegregation event [35] (S1 Text). Because only dividing cells mis-segregate, we have: $T_{p}∼L_{p}^{2}/\left(\beta \lambda \right)$. Similarly, let *T*_*r*_ be the time scale on which the cell population grows: *T*_*r*_ = 1/*r*. If *T*_*p*_ ≪ *T*_*r*_ then extinction via missegregation (hereby “missegregation-induced extinction” or MIE) is possible. The inequality implies that MIE can occur if

$$\begin{array}{c} L_{p}^{2}\ll \frac{\beta \lambda }{r}=\frac{\beta \lambda }{\lambda -\mu }. \end{array}$$ (2)

An important takeaway from this simple argument is that the characteristic scale *L*_*p*_ can play a significant role and is tied to the typical change in DNA content when a missegregation occurs. The narrower the interval of viable DNA content, the weaker the condition, i.e. more combinations of turnover and missegregation rate will exist that lead to MIE. It is interesting to see that in theory, one does not need to increase missegregation. Rather, one can increase birth and death rates in such a way that the quantity (1 − *μ*/λ) decreases. This suggests that tumors with high turnover rates may be more susceptible to MIE.

### 2.2 General discrete model of chromosome mis-segregations

**Fig 1:**
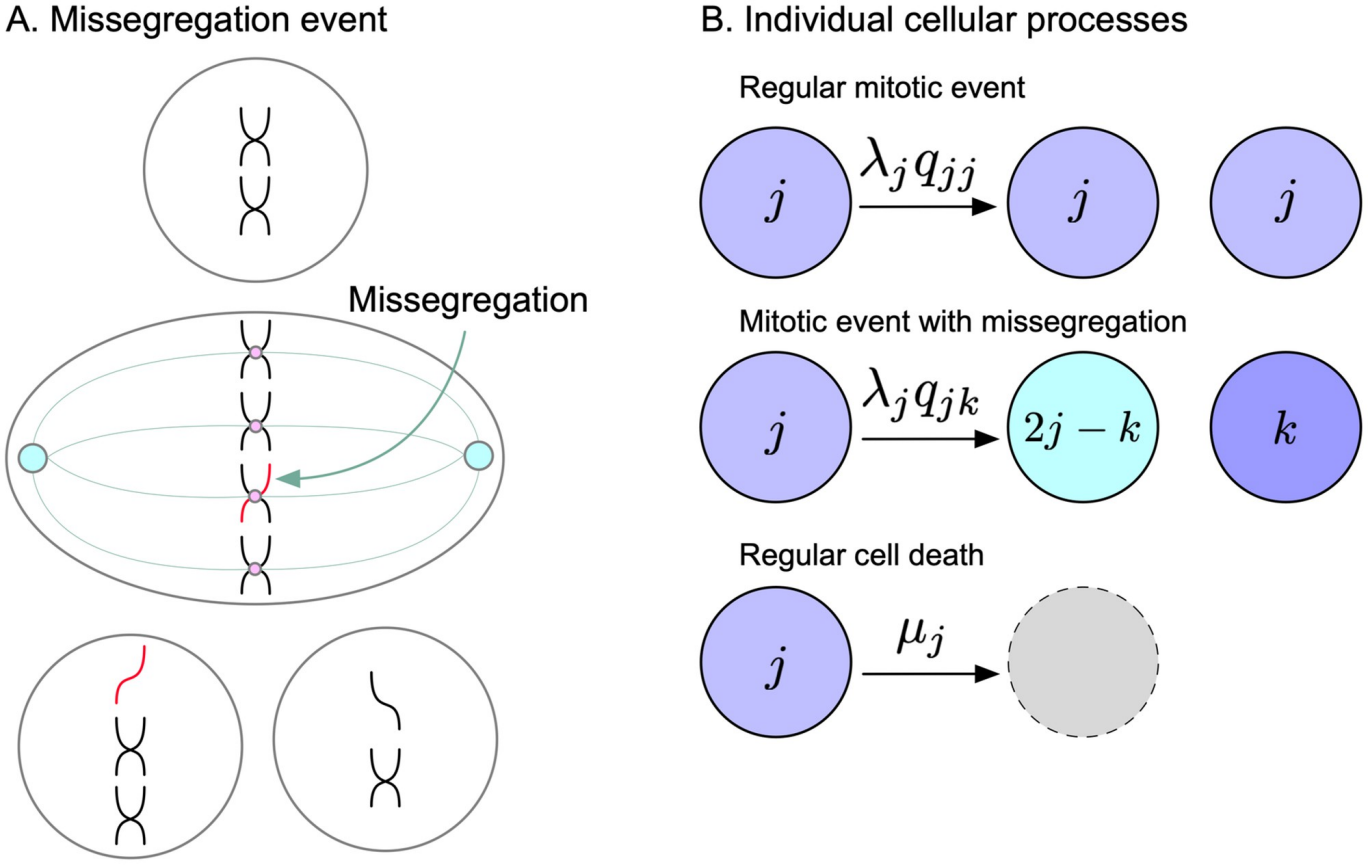
Mathematical modeling of chromosome missegregations. (**A**) Missegregation event. During anaphase, one daughter cell improperly takes both chromosomes leading to aneuploidy. Note the copy number conservation assumption here. (**B**) Model’s individual cellular processes. The tensor *q*_*jk*_ encodes the probability at which a cell with copy number *j* may produce offspring with copy number *k* (and also 2*j* − *k* by copy number conservation). Thus karyotype *j* goes through anaphase with faithful chromosome segregations at a rate λ_*j*_*q*_*jj*_ and missegregates into karyotype *k* (and 2*j* − *k*) at a rate λ_*j*_*q*_*jk*_. Cell death occurs with rate *μ*_*j*_.

Eq (2) was derived under the assumption that shifts in DNA content happen on a continuous scale. But in reality chromosomes are discrete units of information. To investigate whether the impact of turnover rate on MIE remains valid in the discrete setting, we developed a general compartment model that describes the evolution of populations by their karyotypes. Let the *M*-dimensional vector $\vec{i}=\left(i_{1},\ldots i_{M}\right)$ contain the number of copies *i*_*k*_ ≥ 0 of the *k*th component. Two examples are looking at the copy number of whole chromosomes (i.e. *M* = 23) or chromosome arms (i.e. *M* = 46). Movement between the compartments occurs via missegregation. We encapsulate this information in the tensor **q** with non-negative components $q_{\vec{i}\vec{j}}$, which is the probability that division of a cell with karyotype $\vec{i}$ yields a daughter with karyotype $\vec{j}$ (Fig 1). We require a conservation of copy number (which will hold for any resolution). Let $\vec{i}$ be the parent and ${\vec{j}}^{\left(1\right)}$ and ${\vec{j}}^{\left(2\right)}$ be the offspring, then it must be that

$$\begin{array}{c} 2i_{k}=j_{k}^{\left(1\right)}+j_{k}^{\left(2\right)},\;\text{for}\;\text{all}\;k. \end{array}$$ (3)

This imposes structure on **q** since 0 ≤ *j*_*k*_ ≤ 2*i*_*k*_, we must have $q_{\vec{i}{\vec{j}}^{*}}=0$ if *j*_*k*_ > 2*i*_*k*_ for any *k*, thus **q** will be sparse for many applications.

We are now in a position to write a general *M*-dimensional birth-death process:

$$\begin{array}{c} \frac{dn_{\vec{i}}}{dt}=\underset{\text{Inflow}}{\underset{︸}{\sum_{\vec{j}}\lambda_{\vec{j}}n_{\vec{j}}q_{\vec{j}\vec{i}}}}-\underset{\text{outflow}}{\underset{︸}{\lambda_{\vec{i}}n_{\vec{i}}\left(1-q_{\vec{i}\vec{i}}\right)}}-\underset{\text{death}}{\underset{︸}{\mu_{\vec{i}}n_{\vec{i}}}}, \end{array}$$ (4)

where $\lambda_{\vec{i}},\mu_{\vec{i}}$ are the state-dependent birth and death rates, respectively. Eq (3) enters into (4) with the flow rate λ_**q**_. Model parameters are summarized in Table 1.

**Table 1:** Common model parameters used throughout the manuscript.

Parameter	Definition
$\vec{{}_{i}}$	state vector of copy numbers defining a karyotype
$n_{\vec{{}_{i}}}$	cell representation of karyotype $\vec{{}_{i}}$
$q_{\vec{{}_{i}}\vec{{}_{j}}}$	probability that division of a cell with karyotype $\vec{i}$ yields a daughter with karyotype $\vec{j}$
λ	dividing cells /day
*μ*	dying cells /day
*β*	missegregations /chromosome copy /division
*L*_*p*_	characteristic amount of DNA content shifted during a missegregation event
*T*_*p*_	timescale on which missegregations happen
*r*	net growth rate (λ − *μ*)
*T*_*r*_	timescale on which growth happens *T*_*r*_ = 1/*r*

We note that in the absence of missegregation, we require $q_{\vec{i}\vec{j}}=\delta_{\vec{i}\vec{j}}$ where we are using the vector Kronecker delta that is 1 if $\vec{i}={\vec{j}}^{\left(1\right)}={\vec{j}}^{\left(2\right)}$ and 0 otherwise. This uncouples Eq (4) to the classic deterministic birth-death process *dn*_*i*_/*dt* = (λ_*i*_ − *μ*_*i*_)*n*_*i*_ as expected.

We define the shift, *t*, as the net difference in the copy number of a given chromosome type *k*, between the parental cell and its daughter cells. *t* can be positive or negative and accounts for the fact that missegregations can partly or entirely compensate each other. The probability *P*(*t*|*i*_*k*_), that the first daughter cell $j_{k}^{\left(1\right)}$ has a shift of *t* copies, given the parental cell had *i*_*k*_ chromosome copies has been derived by Gusev et al. [32] as:

P(t|ik)=∑odd/even:zk=|t|ik(ikzk)βzk(1-β)ik-zk0.5zk(zkzk-t2), (5)

where missegregations are assumed to be independent and *β* is the missegregation rate per chromosome copy per division. If *t* = 0, both daughter cells will have the same copy number for chromosome *k*. We note that *P*(*t* = 0|*i*_*k*_) can approach 1 only if mis-segregation rate is very low and that ∀*t* ≠ 0: *P*(*t*|*i*_*k*_) ≤ 0.5. If *t* ≠ 0, then there is one cell with *j*_*k*_ = *i*_*k*_ + *t* copies (and another cell with *i*_*k*_ − *t* copies). We can thus calculate the probability that ${\vec{j}}^{\left(1\right)}$ has a specific karyotype:

$$\begin{array}{c} q_{\vec{i}\vec{j}}^{\left(1\right)}=\prod_{k}P\left(i_{k}-j_{k}^{\left(1\right)}|i_{k}\right), \end{array}$$ (6)

where $q_{\vec{i}\vec{j}}^{\left(1\right)}$ is a distribution over possible karyotypes of the first daughter cell, such that $\sum_{\vec{j}}q_{\vec{i}\vec{j}}^{\left(1\right)}=1$. For Eq 4, we require $q_{\vec{i}\vec{j}}$ to be instead a distribution over possible division events. Let $A=P(\vec{j}={\vec{j}}^{\left(1\right)})$ and $B=P(\vec{j}={\vec{j}}^{\left(2\right)})$, so $q_{\vec{i}\vec{j}}=P\left(A\cup B\right)=P\left(A\right)+P\left(B\right)-P\left(A\cap B\right)$. If there is no missegregation ($\vec{i}=\vec{j}$), then *P*(*A*|*B*) = 1 so *P*(*A* ∩ *B*) = *P*(*A*) = *P*(*B*) ⇒ *P*(*A* ∪ *B*) = *P*(*A*) = *P*(*B*). If there is a mis-segregation, *P*(*A* ∩ *B*) = 0 ⇒ *P*(*A* ∪ *B*) = *P*(*A*) + *P*(*B*). Thus:

$$\begin{array}{c} q_{\vec{i}\vec{j}}=\{\begin{array}{cc} \prod_{k}\;P\left(0|i_{k}\right) & \text{if}\;\vec{i}=\vec{j},\\ \prod_{k}\;P\left(i_{k}-j_{k}|i_{k}\right)+\prod_{k}\;P\left(j_{k}-i_{k}|i_{k}\right) & \text{otherwise}. \end{array} \end{array}$$ (7)

If $2\vec{i}=\vec{j_{\left(1\right)}}+\vec{j_{\left(2\right)}}$, then $q_{\vec{i}{\vec{j}}_{\left(1\right)}}=q_{\vec{i}{\vec{j}}_{\left(2\right)}}$, which satisfies the copy number conservation. Further, if we let *β* → 0 we would have:

$$q_{\vec{i}\vec{j}}=\{\begin{array}{cc} 1 & \text{if}\;\vec{i}=\vec{j},\\ 0 & \text{otherwise}, \end{array}$$

which we recognize as the Kronecker delta defined above.

In contrast to the continuum model given in Eq 1, this model takes into account that chromosomes are discrete units of information. We implemented this model in R, allowing numerical simulations of karyotype evolution under variable initial conditions and biological assumptions. Finer genomic resolution leads to more compartments, thereby increasing computational resources required for numerical solutions. The remainder of this manuscript will use the resolution of whole chromosomes to define a karyotype. When missegregation, death and birth rates are independent of karyotype, we will refer to them as *homogeneous*. Conversely, *intra-tumor heterogeneity* in either of these rates will be modeled as a dependency on karyotype. Homogeneous rates imply that these rates will always stay constant over time. Constant rates however do not imply homogeneity within the population, since a heterogeneous but stable karyotype composition will appear constant despite representing multiple rates. In summary, this is a flexible framework, offering the possibility to model a variety of biologically relevant dependencies (e.g. missegregation rate can vary across karyotypes) and variable genomic resolutions.

### 2.3 Chromosomal aggregate model

The model given by Eq (4) is complicated and cumbersome. A simpler model, amendable to analysis involves aggregating all chromosomal data into one index. Alternatively, it can be thought of as focusing on a dosage-sensitive chromosome, that must be present at copy numbers between one and five in order for a cell to survive. We note that this implies that the existence of all but one chromosome is negligible; hence all three terms, “karyotype”, “copy number” and “ploidy” become equivalent. Mathematically, there are many ways to collapse our *M*-dimensional model to 1D, and one such way is to just sum over all indices to get the aggregated number of copies:

$$\begin{array}{c} i=\|\vec{i}{\|}_{1}=\sum_{j}i_{j}. \end{array}$$ (8)

Then our system is given by:

$$\begin{array}{c} \frac{dn_{i}}{dt}=\sum_{j}\;\lambda_{j}n_{j}q_{ji}-\lambda_{i}n_{i}\left(1-q_{ii}\right)-\mu_{i}n_{i}. \end{array}$$ (9)

We will further suppose that the parameters of the model are not dependent on karyotype prevalence (e.g. λ_*i*_ is not dependent on any *n*_*i*_, such as through a carrying capacity or Allee effect etc). This allows us to easily write the Jacobian, which is simply the coefficients of *n*_*j*_ in Eq (9)

$$\begin{array}{c} J_{ij}=\{\begin{array}{cc} \lambda_{i}\left(2q_{ii}-1\right)-\mu_{i} & \text{if}\;i=j,\\ \lambda_{j}q_{ji} & \text{if}\;i\neq j. \end{array} \end{array}$$ (10)

Let [*k*, *K*] with k,K∈N be the interval (not necessarily finite) of viable karyotypes of the aggregate model (Eq (9)). The Jacobian (10) contains information on the local behavior of the system near the extinction state *n*_*i*_ = 0 for all *i*. If all the eigenvalues of the Jacobian at the extinction point are negative, then MIE occurs. The critical curve that separates MIE from exponential growth is when the maximum eigenvalue of *J* is 0.

### 2.4 Ruling out MIE

Here, we establish sufficient conditions for MIE to not occur based on Gershgorin’s circle (GC) theorem [36]. The theorem bounds the locations of the eigenvalues in the complex plane for a given matrix **A**, with elements *a*_*ij*_. The GC theorem stipulates that the eigenvalues must be contained in the circles with centers *a*_*ii*_ and radii *R* = ∑_*i*≠*j*_|*a*_*ij*_|.

Since MIE can be evaded if the maximum eigenvalue exceeds 0, a sufficient condition is that none of the GCs contain a part of the negative reals. Table 2 describes sufficient conditions to avoid MIE for various biological assumptions.

**Table 2:** Sufficient conditions to avoid MIE for various biological scenarios. 1) Only ≤1 chromosome can missegregate per division; all rates are independent of karyotype (i.e. homogeneous). 2) Heterogeneous birth-, death-, and/or missegregation rates. 3) Homogeneous missegregation rate.

Scenario	Sufficient condition to avoid MIE
1. ∀|*i* − *j*| > 1 : *q*_*ij*_ = 0, (*μ*, λ, *β*) constant	$\beta <\beta_{c}=\frac{1}{4}(1-\frac{\mu }{\lambda })$
2. (*μ*, λ, *β*) vary with karyotype *i*	∀*i*: λ_*i*_ (1 − 3*β*_*i*_) − *μ*_*i*_ − λ_*i*+1_*β*_*i*+1_ > 0
3. *β* is constant	$\forall i\text{:}\;\beta <\beta_{c}=\frac{1}{3+\lambda_{i+1}/\lambda_{i}}(1-\frac{\mu_{i}}{\lambda_{i}})$

The general problem for arbitrary **q** can be handled numerically, but analytical conclusions can only be made for specific forms of **q**. The conditions required are given by finding when the GC’s are all contained in the positive half-plane:

$$\begin{array}{cc} & \underset{i}{\text{min}}[\lambda_{i}\left(2q_{ii}-1\right)-\mu_{i}-\lambda_{i}\sum_{j\neq i}q_{ij}]>0, \end{array}$$ (11)

$$\begin{array}{cc} & \underset{i}{\text{min}}[\lambda_{i}\left(2q_{ii}-1\right)-\mu_{i}-\sum_{j\neq i}\lambda_{j}q_{ji}]>0. \end{array}$$ (12)

The conditions imply that MIE cannot happen if any karyotype gains cells faster than it loses cells due to mis-segregations and regular cell death. As both of these need to be positive, we can find the minimum of these, which will provide sufficient condition to escape MIE (see also S1 Text).

### 2.5 Predicting sensitivity to MIE

Given a fixed birth- and death-rate, can we predict at what missegregation rate a population will go extinct? Here we derive critical curves that separate viable from non-viable populations as a function of their turnover- ($\frac{\mu }{\lambda }$) and missegregation rates (*β*). Herein we make three assumptions: (i) all missegregation events are possible (e.g. if parent after S-phase has 2*i* copies, then a daughter cell can have any integer in the range [0, 2*i*]); (ii) homogeneous turnover- and missegregation rates regardless of karyotype; and (iii) that the interval of viable karyotypes [*k*, *K*] is finite. Hereby we consider two types of viable karyotype intervals with different biological interpretations: intervals modeling the copy number of a single individual chromosome (e.g. *k* = 1, *K* = 5) and intervals modeling the ploidy of a cell (e.g. *k* = 22, *K* = 88). The former assumes there exists at least one single critical chromosome for which copy number must stay within a defined range for a cell to be viable. The latter treats all chromosomes as equal and models ploidy as the critical quantity.

To calculate the critical curves conditional on these assumptions, we consider the time evolution of the system given by the matrix form of Eq (9):

$$\begin{array}{c} \frac{dn_{i}}{dt}=n_{i}J, \end{array}$$ (13),

with J defined in Eq (10) and the row vector *n*_*i*_ is the number of cells with copy number state *i*. It is clear that the system reaches a steady state when *n*_*i*_*J* = 0. Nontrivial solutions (i.e. those with nonzero *n*_*i*_) can be found by choosing functions for the mis-segregation rate *β* and death rate *μ* (which parameterise the matrix *J*) such that the dominant eigenvalue is zero (S1 Text). We also simulated the ODE given by Eq (9) until the karyotype distribution reached a steady state (Fig A in S1 Text). Numerical simulations confirmed that the theoretical critical curves separate exponential growth from population extinction (Fig B in S1 Text).

**Fig 2:**
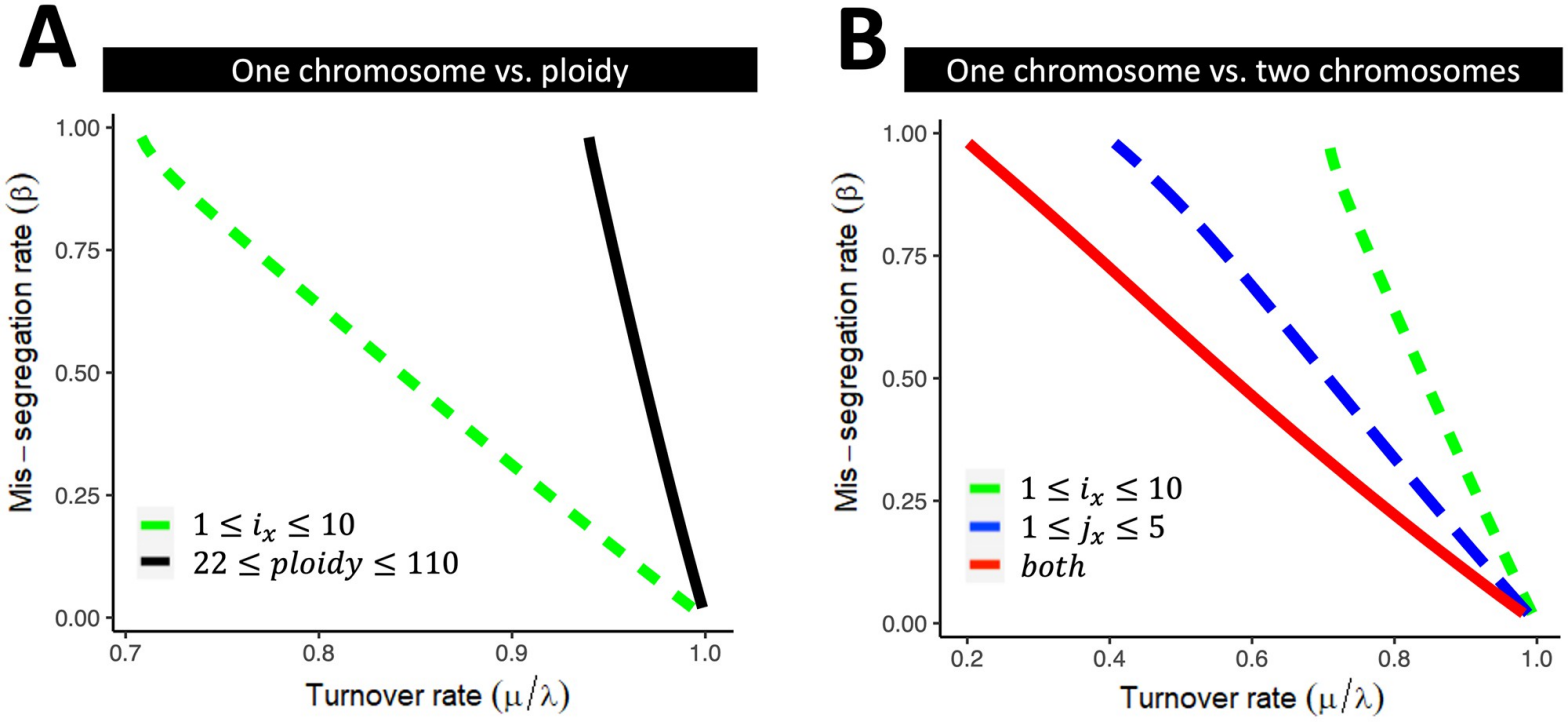
Predicting MIE as a function of homogeneous missegregation and turnover rates. (A-B) Critical curves were obtained by finding ($\beta ,\frac{\mu }{\lambda }$) for which the maximum eigenvalues of the Jacobian (Eq (10)) is 0. (A) We consider two types of viable karyotype intervals with different biological interpretations: intervals modeling the copy number of a single individual chromosome (dashed line) and intervals modeling the ploidy of a cell (solid line). (B) We assume existence of two critical chromosomes *i* and *j*, with intervals of viable karyotypes [*k*_*i*_, *K*_*i*_] and [*k*_*j*_, *K*_*j*_] respectively. We calculate the critical curves assuming cell viability is restricted by only one of the two chromosomes (dashed lines), or by both chromosomes jointly (solid line). Note that the size of the Jaccobian is a function of 1 + *K* − *k*.

We compared scenarios where the viable interval for ploidy is finite to scenarios where the viable interval for the copy number of individual chromosomes is finite (Fig 2A). The latter contracted the viability region considerably more than the former, suggesting MIE due to non-viable copy number of individual chromosome types is more likely than MIE due to non-viable ploidy. When modeling single individual chromosomes, MIE was impossible at low turnover rates, even for very high *β* (Fig 2B). This was because, as *β* → 1, none of the sister chromatids are properly segregated, i.e. they end up in the same cell, resulting in a high representation of cells with an even number of chromosomes. These in turn have a high enough fraction of viable daughter cells, sufficient to keep net growth above 0. In contrast, having more than one chromosome with finite viable karyotype intervals substantially contracted the viability region (Fig 2B), albeit with diminishing costs in viability for each extra chromosome (Fig D in S1 Text).

### 2.6 Quantification of mis-segregation and turnover rates across cancers

When measuring missegregation- and turnover rates in cancers we would expect these rates to lie below the predicted critical curves. To test this we quantified turnover- and missegregation rates at cellular resolution using scRNA-seq data from 15,464 single cells from the TISCH database [37]. Cells originated from 14 tumor biopsies across 12 patients spanning four cancer types across three tissue sites (Lung, Breast and Skin). We leverage the relation between turnover rates of cancers and their respective tissue site of origin [38, 39] (Methods 4.2.3), in order to learn to estimate turnover rate from transcriptomic signatures. A cell’s transcriptome is a channel of information propagation; it is a snapshot of how a cell interacts with and responds to its environment. Transcriptomic signatures have been used to infer various aspects about a tumor, ranging from the level of hypoxia [40], to its cell of origin [41], its mitotic index [42] and other surrogates of cell fitness and risk of disease progression [43, 44]. Cells co-existing in the same tumor, or in the same cell line [45, 46], often differ in their transcriptomes. Together these intra-tumor differences as well as inter-tumor differences in gene expression have the potential to inform how cells and tumors differ in their turnover rates.

**Fig 3:**
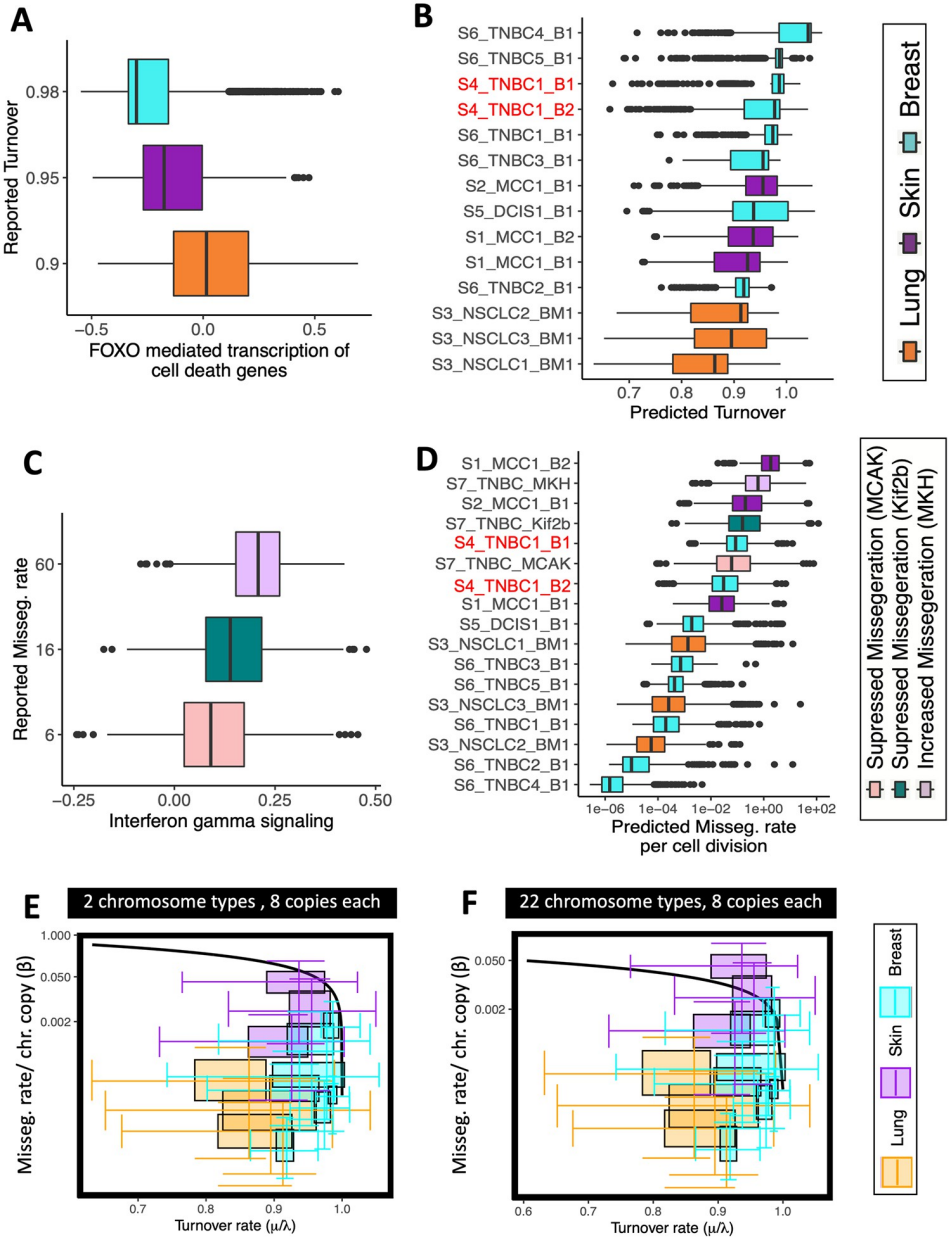
Predicting proximity of a PAN-cancer cohort to MIE. (A-D) Quantification of mis-segregation and turnover rates. (A) Expression of regulators of cell death genes (x-axis) varies across tissue sites (color code) along with turnover rates reported for tumors of the same origin (y-axis; adjusted *R*^2^ = 0.999; *P* = 0.07). (B) A regression model was built from (A) and used to predict turnover rate in 15,464 cells across 14 tumors. (C) Interferon Gamma gene expression (x-axis) measured in 7,879 cells from three human breast cancer cell lines [59] (color code) varies with their % lagging chromosomes quantified from imaging (y-axis; adjusted *R*^2^ = 0.88; *P* = 0.157). (D) A regression model was build from (C) and used to infer turnover rate in 23,343 cells across 17 tumors. (E,F) Empirically inferred missegregation- (*β*), and turnover rates (*μ*/λ) are displayed alongside the theoretical critical MIE curve calculated for two chromosome types (E) and all 22 autosomes (F). The interval of viable karyotypes is between one and eight copies for each chromosome type.

After scRNA-seq data preprocessing (Methods 4.2.1), we performed Gene Set Variation Analysis (GSVA) [47] to quantify the expression activity of 1,629 REACTOME pathways [48] at single cell resolution. For each pathway involved in cell death and apoptosis (12 pathways), we calculated the median expression for a given tissue site and compared it to the median turnover rates [49–58] reported for cancers from the corresponding tissue site (Table A in S1 Text). Of the 12 tested pathways, five had an association with turnover rate (adjusted *R*^2^ ≥ 0.8; Methods 4.2.3), including “*FOXO-mediated transcription of cell death genes*” (Fig 3A, adjusted *R*^2^ > 0.99; *P* = 0.07). We used this pathway signature to estimate turnover rate at single-cell resolution across the 14 tumors (Fig 3B). All but one tumor had predicted turnover rates that were high, but below one (Fig F in S1 Text), consistent with an expanding tumor mass. We note one exception, wherein a pre-treatment breast cancer sample had a median inferred turnover rate of 1.04 (Fig F in S1 Text)—this case was excluded from further analysis.

Given appropriate ground truth data, the same principle can be applied to estimate mis-segregation rates. To estimate mis-segregation rates at cellular resolution we used *Interferon Gamma Signaling* as a surrogate measure of chromosome missegregations [59] (Methods 4.2.4). The rationale for this is that chromosome missegregations can trigger the formation of micronuclei. When micronuclei rupture, their genomic DNA spills into the cytosol. Cytosolic dsDNA is sensed by the cGAS-STING pathway [60], leading to induction of type I interferon stimulated genes [61, 62]. Missegregations lead to the upregulation of interferon production, which in turn subverts lethal epithelial responses to cytosolic DNA. To go from Interferon Gamma expression to mis-segregation rate we integrated aforementioned scRNA-seq dataset with 7,879 transcriptomes sequenced in Bakhoum et al. [59] (Methods 4.2.1). These transcriptomes originated from three cell lines, where members of the kinesin superfamily of proteins were knocked down to increase or decrease mis-segregation rate in a controlled fashion [59]. Live cell imaging of these cells to quantify the resulting missegregation rate was also available [59], allowing for a linear regression model to be fit on this data. As previously reported [59], Interferon Gamma Signaling was correlated to the % lagging chromosomes derived from imaging (Fig 3C; adjusted *R*^2^ = 0.88; *P* = 0.157). The resulting model translates Interferon Gamma expression into units of mis-segregation rate per cell division and was used to estimate mis-segregation rates in the remaining scRNA-seq samples (Fig 3D).

The number of chromosomes a mitotic cell has to segregate among daughter cells varies with ploidy. Therefore, the risk of mis-segregating at least one chromosome should increase with ploidy, rendering the per chromosome missegregation rate a quantity of interest. Calculating the missegregation rate per chromosome requires knowing the karyotype of each cell. To extract this information from the scRNA-seq data we extended an approach we previously described [43] to distinguish chromosome-arms affected by SCNAs from those that are copy number neutral (Methods 4.2.2). The resulting profiles were then clustered into subpopulations of cells with unique karyotypes as previously described [43], allowing for inference of mis-segregation rates per cell division per chromosome for each subpopulation (Fig E in S1 Text).

The variability in missegregation rates and the proximity of turnover rates to homeostasis warrants further investigation into whether increasing missegregation rate is a potential mechanism of extinction in these tumors. We therefore compared missegregation- and turnover rates derived from scRNA-seq data (Fig F in S1 Text) to the critical curves. Since most tumors had high turnover rates, we focused on the critical curves at $\frac{\mu }{\lambda }>0.6$ (Fig 3E and 3F). Of note is the close proximity of the measured rates to the theoretical MIE curves. When imposing between one and eight copies on only two chromosome types, all tumors had median missegregation- and turnover rates that were compatible with our viability predictions (Fig 3E). When imposing between one and eight copies on all 22 autosomes, one of the three skin cancers shifted into the region predicted as non-viable. But for all remaining tumors, even when considering all 22 autosomes, the majority of cells stayed in the viable region (Fig 3F).

### 2.7 Intra-tumor heterogeneity in mis-segregations and turnover

The critical curves shown in Fig 3E and 3F assume a cell population with homogeneous missegregation and turnover rates. The scRNA-seq derived results however suggest that both are likely heterogeneous in reality. We therefore asked whether relaxing this assumption changes the critical curves. To model intra-tumor heterogeneity in missegregation rates, we looked at their relation to ploidy and chromosome copy number. While no significant association between ploidy and turnover rate was evident, the relationship between ploidy and missegregation rate per chromosome per cell division showed a surprising resemblance to the recently hypothesized fitness function of ploidy [63] (Fig G, F in S1 Text). Hereby the commonly observed near-triploid karyotype [34, 64, 65] stands out as a local maximum. A sinus function was therefore chosen to model mis-segregation as a function of ploidy (adjusted *R*^2^ = 0.80; Estimated Variance: 49%, Fig G in S1 Text). A linear association between copy number and missegregation rate was also observed for three of the 22 individual autosomes (adjusted *R*^2^ > 0.1; *P* < < 1*E* − 5, Fig G in S1 Text). The observed relation between missegregation rate per chromosome and ploidy (either of specific chromosomes or in aggregate), is an opportunity to model missegregation rate as a function of ploidy, thereby accounting for intra-tumor heterogeneity.

**Fig 4:**
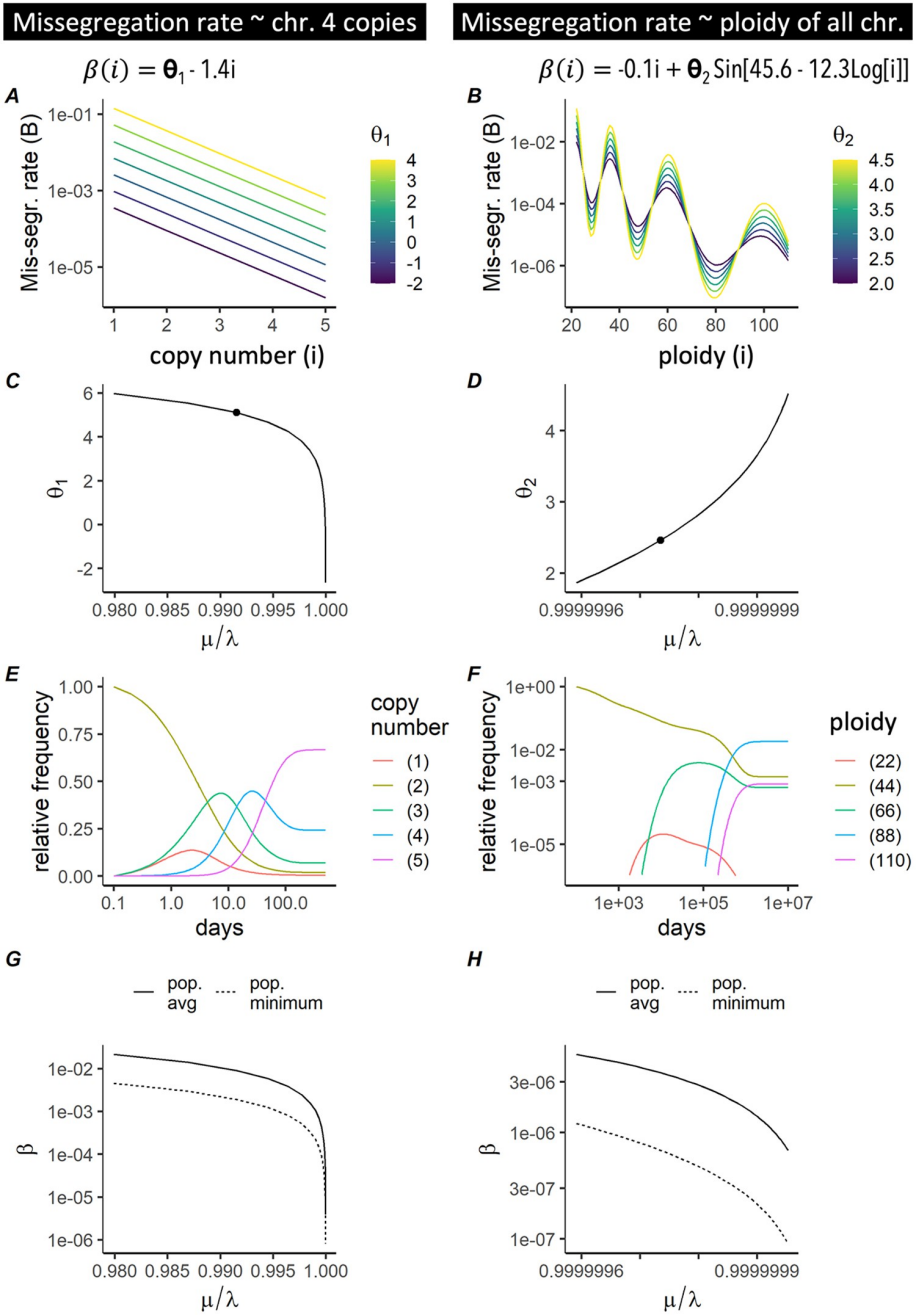
Predicting MIE when missegregation rates are heterogeneous. (A,B) Missegregation rate is modeled as a function of copy number or ploidy (x-axis), with color coded shape parameters *θ*_1_ (A) and *θ*_2_ (B). We consider the copy number of either chromosome 4 alone (A) or of all 22 autosomes in aggregate, i.e. ploidy (B). Varying *θ*_1_, *θ*_2_ yields different missegregation rates (*β*). (C) Critical curve was obtained for (A) by finding ($\theta_{1},\frac{\mu }{\lambda }$) for which the maximum eigenvalues of the Jacobian (Eq (10)) is 0. (D) Critical curve was obtained for (B) in the same manner as in (C), but here equations were solved for ($\theta_{2},\frac{\mu }{\lambda }$). (E,F) We used the parameters highlighted in (C,D) to simulate missegregations until the karyotype composition reached a steady state. (G,H) The eigenvectors corresponding to the eigenvalues found in C/D are the steady state karyotype proportions. These are used in conjunction with the kernels in A/B to determine the population average and minimum missegregation rates (y-axis).

Modeling missegregation rate as linear or sinusoidal functions of the copy number of chromosome 4 and overall ploidy respectively (Fig 4A and 4B), we calculated how parameters of both functions shape the critical curve (Fig 4; Fig A in S1 Text). In both cases the population evolved toward the karyotype with the lowest mis-segregation rate (Fig 4E and 4F). Unless turnover rates are exceedingly high, this convergence to the global minimum mis-segregation rates effectively rendered MIE impossible (Fig 4G and 4H). The absolute value of that minimum explains the difference in the location of the critical curve between the two scenarios (Fig 4G and 4H), and the overall low risk of MIE in large cell populations.

If we also model heterogeneous death rates, the interplay between mis-segregation and death rate, rather than the minimum mis-segregation rate alone, determine whether MIE will occur. More generally, when both missegregation- and death rates are heterogeneous, a necessary condition for MIE is that karyotypes with low mis-segregation rates must also have high death rates (Fig C in S1 Text). This condition is exactly identical to the sufficient condition for ruling out MIE described by Eq (11), and was corroborated by combinations of kernels of missegregation- and death rate (Fig C in S1 Text). Taken together these results suggests that heterogeneous missegregation rates can protect a population from extinction.

## 3 Discussion

We have presented a general approach for modeling whole chromosome missegregations, including a deterministic mathematical framework and scRNA-seq analysis methods to infer effective model parameters. In contrast to prior models of chromosome missegregations [31–33], our model does not rest on the assumption that the fitness effect of a mis-segregation is the same, regardless of the karyotype context in which it happens. This feature offers the flexibility to identify potential synergies between copy number changes of multiple chromosomes [66]. A second difference to prior models of missegregations [31], is the decoupling of a cell’s life cycle from the life cycle of individual chromosomes. This allows simulating intra-tumor heterogeneity in mis-segregation, death- and proliferation rates across cells, which can manifest as temporal variations in these rates (when the karyotype composition changes over time).

Theoretical analysis of the mathematical model has shown the existence of a potential mechanism of tumor control through the region in parameter space we have called MIE. As a first application of the model we have thus focused on the identification of critical curves that separate viable populations from MIE, as a function of their turnover- and missegregation rates. A central assumption of these calculations is that cells are not viable unless they carry a certain number of copies of a given chromosome, that must lie within a predefined interval. To our knowledge, Fig 2 contains the first predictions of MIE that consider viable karyotype intervals of multiple chromosomes simultaneously, as well as the turnover rate of the population. We compared these theoretical critical curves to missegregation- and turnover rates inferred from scRNA-seq of 13 tumors across four cancer types from three tissue sites. The majority of tumors across all tissue sites studied had missegregation- and turnover rates that were compatible with our viability predictions (Fig 3E and 3F). This remained true when a dependency of missegregation rates on ploidy was introduced. In populations with heterogeneous mis-segregation rates, the subpopulation with the minimum mis-segregation rate protects the population from extinction (Fig 4G and 4H). Our results emphasize that large, heterogeneous tumors have an inbuilt protection from MIE. That each tumor consists of cells with heterogeneous missegregation rates, the measurement being just the population-average rate, is a likely scenario supported by recent results [43, 67]. Karyotypes associated with low missegregation rates act as a stabilizing refuge, protecting the population from extinction. Intra-tumor heterogeneity, including heterogeneity in missegregation rates, increases as tumors progress. Our predictions suggest that this intra-tumor heterogeneity renders MIE unlikely.

The model raises some important theoretical questions related to malignant and non-malignant cells. In particular, it is well known that normal cells maintain a level of homeostasis through a balanced turnover rate *μ*/λ ≈ 1. This seems to imply that *all* normal cells lay at the MIE boundary and are even more sensitive to MIE than malignant cells. Any finite missegregation rate would thus lead to the slow removal of normal cells over time. There are two potential explanations for this behavior. A likely explanation is given by our assumption that the birth rate is independent of population size. It is easy to see that introducing dependency on the total populations size (e.g. carrying capacity) could alleviate this issue as cell death would increase the birth rate in order to return to homeostasis. An alternative explanation is that this is just another natural aging mechanism through which normal cells are slowly displaced. Transformed cells often lose the homeostatic control mechanisms and so are likely less susceptible to contact inhibition.

Limitations of our approach include unknown precision of mis-segregation and turnover rates inferred from scRNA-seq. In line with prior reports [68], mis-segregation rates were higher in higher stage cancer, while normal tissue used as control had the lowest mis-segregation rates (Fig I in S1 Text). The number of subpopulations with distinct karyotypes was also by trend higher in late stage tumors—a finding that is also consistent with prior reports [68–70]. Other limitations include those of ordinary differential equation models, such as the lack of stochasticity, rendering all conclusions valid only for large populations, where all karyotypes are accessible and have cellular representation. Understanding how missegregations shape extinction events in early stage cancers would require a different approach. Our model does not explicitly account for several biological mechanisms which are relevant to karyotype evolution, including WGD, missegregation induced apoptosis in the subsequent G1 phase of the cell cycle, and the formation of micronuclei. Extensions to model these phenomena are discussed in S1 Text.

We and others have previously defined an adaptive fitness landscape as a genotype-fitness map, which associates to each karyotype a fitness value [71, 72]. Its size and context dependency (e.g. on the tumor microenvironment) renders reconstruction of fitness landscapes a challenging task. Nevertheless, recent efforts have linked specific karyotypes to differences in cell fitness [73], albeit under several simplifying, and partly unrealistic assumptions (e.g. neglecting epistasis). The mathematical model presented here offers the flexibility necessary to begin reconstructing adaptive fitness landscapes. Future applications of this model will also include studying the fitness costs and benefits of high ploidy. Coexistence of cancer cells at opposite extremes of the ploidy spectrum occurs frequently in cancer and missegregations are a major contributor to heterogeneous ploidy states within a population. Our model can help understand how much robustness high ploidy confers to the sudden genome-dosage imbalance caused by a missegregation event [12] and can help quantify the energetic requirements of high ploidy cells. Modeling intra-tumor heterogeneity in mis-segregation rates and their effect on karyotype evolution over hundreds of generations can reveal how selection acts upon coexisting karyotypes, as a powerful tool for genotype-to-phenotype mapping in various microenvironments.

## 4 Materials and methods

### 4.1 Numerical Simulations

Numerical simulations were performed for a range of input parameters (*β*, *μ*) to validate the predicted critical curves. All simulations had a uniform diploid population as initial condition. Each simulation ran until a quasi-steady-state (QSS) had been reached, considered to occur when the rate of change in karyotype composition was less than 0.1%/day (although the cell population may still be growing or shrinking—therefore “quasi”). Upon satisfaction of this condition, simulations with a positive rate of change in the total cell population were considered to be in the exponential growth regime.

In order to determine the population average missegregation rates *β*_*pop*_ and death rates *μ*_*pop*_ which are viable QSS’s for the system, we performed numerical simulations for each pairwise combination of missegregation- and death rate kernels—*β*(*i*, *B*) and *μ*(*i*, *M*) respectively (Fig C in S1 Text). For each pairwise combination of kernels, simulations were performed using large, manually curated ranges for the input parameters (*B*, *M*), before *β*_*pop*_ and *μ*_*pop*_ were calculated based on the QSS reached by the system. Population average missegregation rate (*β*_*pop*_) was defined as fraction of divisions in which a missegregation occurs (i.e. 1 − (1 − *β*)^*i*^).

Numerical simulations were performed using R, all code is available on Github.

### 4.2 Quantification of ploidy, missegregation- and turnover rates across cancers

To parametrize our ODE, we derive ploidy, mis-segregation and turnover rates from an integrated scRNA-seq dataset. Fourteen samples from 12 patients across four cancer types were downloaded from the TISCH database [37] and analyzed as follows.

#### 4.2.1 scRNA-Seq data integration

Filtered gene-barcodes matrices containing only barcodes with UMI counts passing threshold for cell detection were imported to Seurat v4.0 for downstream analysis. Barcodes with fewer than 500 genes expressed or more than 25% mitochondrial UMIs were filtered out; genes expressed in fewer than 3 barcodes were also excluded. Raw counts of different datasets were merged using merge function. Standard library size and log-normalization was performed on raw UMI counts using NormalizeData, and top 5000 most variable genes were identified by the “vst” method in FindVariableFeatures. S and G2/M cell cycle phase scores were assigned to cells based on previously defined gene sets(9) using CellCycleScoring function. Normalized UMI counts were further scaled using ScaleData function by regressing against total reads count, % of mitochondrial UMIs, and cell cycle phase scores (G2M.Score, and S.Score) to mitigate the effects of sequencing depth and cell cycle heterogeneity. UMAP analysis of the data after these normalization steps were performed shows that cells cluster by cell types, rather than by study, which indicated that the batch effects were delimited by normalization and scaling (Fig H in S1 Text). Because 10X measures UMI (copy of transcripts), not number of reads mapped to genes and because GSVA uses the ranking of the transcripts, the data from different experiments are comparable when quantified into pathway activity.

#### 4.2.2 Estimating ploidy from scRNA-Seq

Our goal was to distinguish chromosome(-arm)s affected by SCNAs from those that are copy number neutral, given a set of tumor cells and normal cells from same patient. Normal cells (often immune cells) were not of the same type as tumor cells (epithelial). Hence, using them directly as a control to calculate absolute copy number in tumor cells is problematic: immune cells express different numbers of genes (often less), and may have a different viability during scRNA-seq library preparation. To overcome this challenge, we assume that at least one chromosome is diploid in all tumor cells and that most SCNAs are clonal (i.e. they affect all tumor cells) [74].

We first sort chromosomes by the p-value of differential chromosome-specific gene expression between tumor and normal cells in descending order of significance. We chose an *x* ∈ {1..22} and define $\vec{i}$ and $\vec{j}$ as the vectors of the first *x* and last (22 − *x*) chromosomes in the sorted set respectively. We then proceed as follows: (i) We assume all chromosomes in $\vec{i}$ have identical (diploid) copy number in tumor and normal cells. The average ratio of expression between tumor and normal cells for these *x* chromosomes should thus be 1. Deviation from 1 is the bias (*ϵ*_*x*_) we estimate between tumor and normal cells: $\epsilon_{x}=1-tumor_{\vec{i}}/normal_{\vec{i}}$. (ii) We calculate the vector $\vec{j}$ of copy numbers of chromosomes with SCNAs (all except the first *x*), with entries *j*_*k*_ as: *j*_*k*_ = (2/*ϵ*_*x*_) * (*tumor*_*k*_/*normal*_*k*_) for each chromosome *k*. (iii) We evaluate deviation of $\vec{j}$ from the closest integers: $E_{x}=\frac{1}{\|\vec{j}\|}\sum_{k\in \vec{j}}{\left(i_{k}-\lfloor i_{k}\rceil \right)}^{2}$. Repeating steps (i-iii) for all possible values of *x* lets us choose the x* ≔ arg min_*x*_ *E_x_* (Fig J in S1 Text). This classifies the last (22 − x*) chromosomes as chromosomes affected by SCNAs and gives us their absolute copy numbers in the respective $\vec{j}$.

We then used LIAYSON [43] to classify cells into subpopulations with distinct karyotypes. The number of subpopulations was by trend higher in tumors of high ploidy (Spearman *r* = 0.484; *P* = 0.079), but lower in tumors with high turnover rates (Spearman *r* = −0.489; *P* = 0.076; Fig F in S1 Text). We also observed that a clone’s ploidy was positively associated with its variance in turnover rates (Spearman *r* = 0.41; *P* = 7.9*E* − 5). This positive association was also observed when considering each of the three tissue sites (Breast, Lung, Skin) individually, albeit it only reached significance in Lung cancer (Spearman *r* = 0.63; *P* = 8.1*E* − 6).

#### 4.2.3 Estimating turnover rates from scRNA-seq

Reported proliferation rates from tumors correlate to turnover rates from their respective normal tissue of origin (Pearson *r* = 0.93, *P* = 0.021; Table A in S1 Text). The same is true for reported cancer cell death rates, which also correlate to the death rates of their tissue of origin (Pearson *r* = 0.92, *P* = 0.025; Table A in S1 Text). The relation between turnover rates of cancers and their respective tissue site of origin [38, 39], is an opportunity to learn how to read these rates from transcriptomic signatures. We performed Gene Set Variation Analysis (GSVA) [47] to quantify the expression activity of 1,629 REACTOME pathways [48] in a cumulative total of 43,596 single cells from 15 samples across three tissue sites. For each pathway involved in cell death and apoptosis (12 pathways), we calculated the average expression among all cells of a given tissue site and used it to model the median turnover rates [49–58, 75] reported for cancers from the corresponding tissue site (Table A in S1 Text):

We fitted a linear regression model on the combined dataset as follows:

$$\begin{array}{c} \tau =a*x+b, \end{array}$$ (14)

where *x* is the average pathway expression signature per cancer and *τ* is the turnover rate reported in literature for that cancer type. Of all tested pathways, five had an association with turnover rate (adjusted *R*^2^ ≥ 0.8), including “*FOXO-mediated transcription of cell death genes*” (adjusted *R*^2^ = 0.999; *P* = 0.07). This pathway signature was then used to estimate *τ* in each single cell across the four cancer types (Fig 3B). We set birth rate to 1, and used *μ* ≔ *τ* as death rate for all further mathematical modeling.

#### 4.2.4 Estimating missegregation rate from scRNA-seq

Interferon Signaling has been proposed as potential surrogate measure for CIN [59]. To predict missegregation rate from expression of genes involved in *Interferon Gamma Signaling*, we used a similar approach as for turnover rate. We fitted a linear regression on the breast cancer data from [59] as follows:

$$\begin{array}{c} \beta =a\gamma +b, \end{array}$$ (15)

where *β* is the log2 of observed percentage of cells with lagging chromosomes and *γ* the Interferon Gamma Signaling activity as quantified with GSVA (adjusted R-square = 0.999, p-value = 0.0103; Fig 3C). The resulting model was then used to predict missegregation rate in 15,464 single cells from the 14 tumor samples (Fig 3D). We divided the predicted missegregation rate by ploidy to obtain *β* for all further mathematical modeling.

The hereby obtained relationship between missegregation rate and karyotype (Fig G in S1 Text), was similar to how karyotype and fitness are thought to be linked [63]. Namely, triploid karyotypes had higher mis-segregation rates and missegregation rates of euploid states tended to decrease with ploidy. This trend was only evident when looking at the ploidy spectrum across all four cancer types. Variability in ploidy was too low to test if this observation holds across tumors of a given type and especially across subpopulations within a given tumor. That ploidy and cancer type are confounded prevents any causal conclusions to be drawn from this analysis. This correlation is however to be expected, because ploidy is highly cancer types specific [71, 76, 77].

## Supporting information

S1 Text Supplementary methods, figures and tables. (PDF)

## References

[1] Choma D, Daures J, Quantin X, Pujol J. Aneuploidy and prognosis of non-small-cell lung cancer: a meta-analysis of published data. British journal of cancer. 2001;85(1):14–22. doi: 10.1054/bjoc.2001.1892 11437396 PMC2363907

[2] Donepudi MS, Kondapalli K, Amos SJ, Venkanteshan P, others. Breast cancer statistics and markers. Journal of cancer research and therapeutics. 2014;10(3):506. 25313729 10.4103/0973-1482.137927

[3] Walther A, Houlston R, Tomlinson I. Association between chromosomal instability and prognosis in colorectal cancer: a meta-analysis. Gut. 2008;57(7):941–950. doi: 10.1136/gut.2007.135004 18364437

[4] Lennartz M, Minner S, Brasch S, Wittmann H, Paterna L, Angermeier K, et al. The combination of DNA ploidy status and PTEN/6q15 deletions provides strong and independent prognostic information in prostate cancer. Clinical Cancer Research. 2016;22(11):2802–2811. doi: 10.1158/1078-0432.CCR-15-0635 26813356

[5] Stieber D, Golebiewska A, Evers L, Lenkiewicz E, Brons NH, Nicot N, et al. Glioblastomas are composed of genetically divergent clones with distinct tumourigenic potential and variable stem cell-associated phenotypes. Acta neuropathologica. 2014;127(2):203–219. doi: 10.1007/s00401-013-1196-4 24154962 PMC3895194

[6] Thompson SL, Compton DA. Examining the link between chromosomal instability and aneuploidy in human cells. The Journal of Cell Biology. 2008;180(4):665–672. doi: 10.1083/jcb.200712029 18283116 PMC2265570

[7] Bakhoum SF, Kabeche L, Murnane JP, Zaki BI, Compton DA. DNA-damage response during mitosis induces whole-chromosome missegregation. Cancer Discovery. 2014;4(11):1281–1289. doi: 10.1158/2159-8290.CD-14-0403 25107667 PMC4221427

[8] Hayashi MT, Cesare AJ, Fitzpatrick JAJ, Lazzerini-Denchi E, Karlseder J. A telomere-dependent DNA damage checkpoint induced by prolonged mitotic arrest. Nature Structural & Molecular Biology. 2012;19(4):387–394. doi: 10.1038/nsmb.2245 22407014 PMC3319806

[9] Pedersen RT, Kruse T, Nilsson J, Oestergaard VH, Lisby M. TopBP1 is required at mitosis to reduce transmission of DNA damage to G1 daughter cells. The Journal of Cell Biology. 2015;210(4):565–582. doi: 10.1083/jcb.201502107 26283799 PMC4539992

[10] Minocherhomji S, Ying S, Bjerregaard VA, Bursomanno S, Aleliunaite A, Wu W, et al. Replication stress activates DNA repair synthesis in mitosis. Nature. 2015;528(7581):286–290. doi: 10.1038/nature16139 26633632

[11] Bakhoum SF, Kabeche L, Compton DA, Powell SN, Bastians H. Mitotic DNA Damage Response: At the Crossroads of Structural and Numerical Cancer Chromosome Instabilities. Trends in Cancer. 2017;3(3):225–234. doi: 10.1016/j.trecan.2017.02.001 28718433 PMC5518619

[12] Sheltzer JM, Ko JH, Replogle JM, Burgos NCH, Chung ES, Meehl CM, et al. Single-chromosome Gains Commonly Function as Tumor Suppressors. Cancer Cell. 2017;31(2):240–255. doi: 10.1016/j.ccell.2016.12.004 28089890 PMC5713901

[13] Ganem NJ, Godinho SA, Pellman D. A mechanism linking extra centrosomes to chromosomal instability. Nature. 2009;460(7252):278–282. doi: 10.1038/nature08136 19506557 PMC2743290

[14] Ertych N, Stolz A, Stenzinger A, Weichert W, Kaulfuß S, Burfeind P, et al. Increased microtubule assembly rates influence chromosomal instability in colorectal cancer cells. Nature Cell Biology. 2014;16(8):779–791. doi: 10.1038/ncb2994 24976383 PMC4389786

[15] Bakhoum SF, Thompson SL, Manning AL, Compton DA. Genome stability is ensured by temporal control of kinetochore-microtubule dynamics. Nature Cell Biology. 2009;11(1):27–35. doi: 10.1038/ncb1809 19060894 PMC2614462

[16] Bakhoum SF, Compton DA. Kinetochores and disease: keeping microtubule dynamics in check! Current Opinion in Cell Biology. 2012;24(1):64–70. doi: 10.1016/j.ceb.2011.11.012 22196931 PMC3294090

[17] Bates D, Eastman A. Microtubule destabilising agents: far more than just antimitotic anticancer drugs. British Journal of Clinical Pharmacology. 2017;83(2):255–268. doi: 10.1111/bcp.13126 27620987 PMC5237681

[18] Leopardi P, Marcon F, Dobrowolny G, Zijno A, Crebelli R. Influence of donor age on vinblastine-induced chromosome malsegregation in cultured peripheral lymphocytes. Mutagenesis. 2002;17(1):83–88. doi: 10.1093/mutage/17.1.83 11752239

[19] Lee HS, Lee NCO, Kouprina N, Kim JH, Kagansky A, Bates S, et al. Effects of Anticancer Drugs on Chromosome Instability and New Clinical Implications for Tumor-Suppressing Therapies. Cancer Research. 2016;76(4):902–911. doi: 10.1158/0008-5472.CAN-15-1617 26837770 PMC4827779

[20] Bakhoum SF, Kabeche L, Wood MD, Laucius CD, Qu D, Laughney AM, et al. Numerical chromosomal instability mediates susceptibility to radiation treatment. Nature Communications. 2015;6:5990. doi: 10.1038/ncomms6990 25606712 PMC4516720

[21] Dai C, Sun F, Zhu C, Hu X. Tumor environmental factors glucose deprivation and lactic acidosis induce mitotic chromosomal instability-an implication in aneuploid human tumors. PLoS One. 2013;8(5):e63054. doi: 10.1371/journal.pone.0063054 23675453 PMC3651256

[22] Kondoh M, Ohga N, Akiyama K, Hida Y, Maishi N, Towfik AM, et al. Hypoxia-induced reactive oxygen species cause chromosomal abnormalities in endothelial cells in the tumor microenvironment. PloS one. 2013;8(11):e80349. doi: 10.1371/journal.pone.0080349 24260373 PMC3829944

[23] Dewhurst SM, McGranahan N, Burrell RA, Rowan AJ, Grönroos E, Endesfelder D, et al. Tolerance of whole-genome doubling propagates chromosomal instability and accelerates cancer genome evolution. Cancer Discovery. 2014;4(2):175–185. doi: 10.1158/2159-8290.CD-13-0285 24436049 PMC4293454

[24] Fujiwara T, Bandi M, Nitta M, Ivanova EV, Bronson RT, Pellman D. Cytokinesis failure generating tetraploids promotes tumorigenesis in p53-null cells. Nature. 2005;437(7061):1043–1047. doi: 10.1038/nature04217 16222300

[25] Vitale M. Intratumor BRAFV600E heterogeneity and kinase inhibitors in the treatment of thyroid cancer: a call for participation. Thyroid: official journal of the American Thyroid Association. 2013;23(4):517–519. doi: 10.1089/thy.2012.0614 23398043

[26] Bakhoum SF, Compton DA. Chromosomal instability and cancer: a complex relationship with therapeutic potential. The Journal of Clinical Investigation. 2012;122(4):1138–1143. doi: 10.1172/JCI59954 22466654 PMC3314464

[27] Thompson SL, Compton DA. Proliferation of aneuploid human cells is limited by a p53-dependent mechanism. The Journal of Cell Biology. 2010;188(3):369–381. doi: 10.1083/jcb.200905057 20123995 PMC2819684

[28] Santaguida S, Richardson A, Iyer DR, M’Saad O, Zasadil L, Knouse KA, et al. Chromosome mis-segregation generates cell cycle-arrested cells with complex karyotypes that are eliminated by the immune system. Developmental cell. 2017;41(6):638–651.e5. doi: 10.1016/j.devcel.2017.05.022 28633018 PMC5536848

[29] Roylance R, Endesfelder D, Gorman P, Burrell RA, Sander J, Tomlinson I, et al. Relationship of extreme chromosomal instability with long-term survival in a retrospective analysis of primary breast cancer. Cancer epidemiology, biomarkers & prevention: a publication of the American Association for Cancer Research, cosponsored by the American Society of Preventive Oncology. 2011;20(10):2183–2194. doi: 10.1158/1055-9965.EPI-11-0343 PMC3199437 21784954

[30] Birkbak NJ, Eklund AC, Li Q, McClelland SE, Endesfelder D, Tan P, et al. Paradoxical relationship between chromosomal instability and survival outcome in cancer. Cancer research. 2011;71(10):3447–3452. doi: 10.1158/0008-5472.CAN-10-3667 21270108 PMC3096721

[31] Gusev Y, Kagansky V, Dooley WC. A stochastic model of chromosome segregation errors with reference to cancer cells. Mathematical and Computer Modelling. 2000;32(1):97–111. doi: 10.1016/S0895-7177(00)00122-9

[32] Gusev Y, Kagansky V, Dooley WC. Long-term dynamics of chromosomal instability in cancer: A transition probability model. Mathematical and Computer Modelling. 2001;33(12):1253–1273. doi: 10.1016/S0895-7177(00)00313-7

[33] Elizalde S, Laughney AM, Bakhoum SF. A Markov chain for numerical chromosomal instability in clonally expanding populations. PLoS computational biology. 2018;14(9):e1006447. doi: 10.1371/journal.pcbi.1006447 30204765 PMC6150543

[34] Laughney AM, Elizalde S, Genovese G, Bakhoum SF. Dynamics of Tumor Heterogeneity Derived from Clonal Karyotypic Evolution. Cell Reports. 2015;12(5):809–820. doi: 10.1016/j.celrep.2015.06.065 26212324

[35] Cantor B. Fick’s Laws: Diffusion. In: Cantor B, editor. The Equations of Materials. Oxford University Press; 2020. p. 0. Available from: 10.1093/oso/9780198851875.003.0007.

[36] Varga RS. Geršgorin and his circles. vol. 36. Springer Science & Business Media; 2010.

[37] Sun D, Wang J, Han Y, Dong X, Ge J, Zheng R, et al. TISCH: a comprehensive web resource enabling interactive single-cell transcriptome visualization of tumor microenvironment. Nucleic Acids Research. 2021;49(D1):D1420–D1430. doi: 10.1093/nar/gkaa1020 33179754 PMC7778907

[38] Couturier CP, Ayyadhury S, Le PU, Nadaf J, Monlong J, Riva G, et al. Single-cell RNA-seq reveals that glioblastoma recapitulates a normal neurodevelopmental hierarchy. Nature Communications. 2020;11(1):3406. doi: 10.1038/s41467-020-17186-5 32641768 PMC7343844

[39] Li R, Liao B, Wang B, Dai C, Liang X, Tian G, et al. Identification of Tumor Tissue of Origin with RNA-Seq Data and Using Gradient Boosting Strategy. BioMed Research International. 2021;2021:6653793. doi: 10.1155/2021/6653793 33681364 PMC7904362

[40] Beig N, Patel J, Prasanna P, Hill V, Gupta A, Correa R, et al. Radiogenomic analysis of hypoxia pathway is predictive of overall survival in Glioblastoma. Scientific Reports. 2018;8(1):7. doi: 10.1038/s41598-017-18310-0 29311558 PMC5758516

[41] Xu Q, Chen J, Ni S, Tan C, Xu M, Dong L, et al. Pan-cancer transcriptome analysis reveals a gene expression signature for the identification of tumor tissue origin. Modern Pathology. 2016;29(6):546–556. doi: 10.1038/modpathol.2016.60 26990976

[42] Yang Z, Wong A, Kuh D, Paul DS, Rakyan VK, Leslie RD, et al. Correlation of an epigenetic mitotic clock with cancer risk. Genome Biology. 2016;17:205. doi: 10.1186/s13059-016-1064-3 27716309 PMC5046977

[43] Andor N, Lau BT, Catalanotti C, Sathe A, Kubit MA, Chen J, et al. Joint single cell DNA-seq and RNA-seq of gastric cancer cell lines reveals rules of in vitro evolution. NAR Genomics and Bioinformatics. 2020;. doi: 10.1093/nargab/lqaa016 32215369 PMC7079336

[44] Verhaak RGW, Hoadley KA, Purdom E, Wang V, Qi Y, Wilkerson MD, et al. Integrated genomic analysis identifies clinically relevant subtypes of glioblastoma characterized by abnormalities in PDGFRA, IDH1, EGFR, and NF1. Cancer cell. 2010;17(1):98–110. doi: 10.1016/j.ccr.2009.12.020 20129251 PMC2818769

[45] Ben-David U, Siranosian B, Ha G, Tang H, Oren Y, Hinohara K, et al. Genetic and transcriptional evolution alters cancer cell line drug response. Nature. 2018;560(7718):325–330. doi: 10.1038/s41586-018-0409-3 30089904 PMC6522222

[46] Ben-David U, Ha G, Tseng YY, Greenwald NF, Oh C, Shih J, et al. Patient-derived xenografts undergo mouse-specific tumor evolution. Nature Genetics. 2017;49(11):1567–1575. doi: 10.1038/ng.3967 28991255 PMC5659952

[47] Hänzelmann S, Castelo R, Guinney J. GSVA: gene set variation analysis for microarray and RNA-Seq data. BMC Bioinformatics. 2013;14(1):7. doi: 10.1186/1471-2105-14-7 23323831 PMC3618321

[48] Croft D, Mundo AF, Haw R, Milacic M, Weiser J, Wu G, et al. The Reactome pathway knowledgebase. Nucleic Acids Research. 2014;42(Database issue):D472–D477. doi: 10.1093/nar/gkt1102 24243840 PMC3965010

[49] Rew D, Wilson G. Cell production rates in human tissues and tumours and their significance. Part II: clinical data. European Journal of Surgical Oncology (EJSO). 2000;26(4):405–417. doi: 10.1053/ejso.1999.0907 10873364

[50] Haustermans K, Vanuytsel L, Geboes K, Lerut T, Van Thillo J, Leysen J, et al. In vivo cell kinetic measurements in human oesophageal cancer: what can be learned from multiple biopsies? European Journal of Cancer. 1994;30(12):1787–1791. doi: 10.1016/0959-8049(94)00252-Z 7880607

[51] Choi SJ, Kim HS, Ahn SJ, Jeong YM, Choi HY. Evaluation of the growth pattern of carcinoma of colon and rectum by MDCT. Acta Radiologica. 2013;54(5):487–492. doi: 10.1177/0284185113475923 23436826

[52] IDE M, Jimbo M, Yamamoto M, Umebara Y, Hagiwara S, Kubo O. Growth rate of intracranial meningioma: tumor doubling time and proliferating cell nuclear antigen staining index. Neurologia medico-chirurgica. 1995;35(5):289–293. doi: 10.2176/nmc.35.289 7623949

[53] Bolin S, Nilsson E, Sjödahl R. Carcinoma of the colon and rectum-growth rate. Annals of surgery. 1983;198(2):151. doi: 10.1097/00000658-198308000-00007 6870372 PMC1353072

[54] Margolese R, HG BT. Natural history and prognostic markers. Holland-Frei Cancer Medicine 6th edition ed Hamilton (ON): BC Decker. 2003;.

[55] Zharinov G, Gushchin V. The rate of tumor growth and cell loss in cervical cancer. Voprosy onkologii. 1989;35(1):21–25. 2919502

[56] Carlson JA. Tumor doubling time of cutaneous melanoma and its metastasis. The American journal of dermatopathology. 2003;25(4):291–299. doi: 10.1097/00000372-200308000-00003 12876486

[57] Harris K, Khachaturova I, Azab B, Maniatis T, Murukutla S, Chalhoub M, et al. Small cell lung cancer doubling time and its effect on clinical presentation: a concise review. Clinical Medicine Insights: Oncology. 2012;6:CMO–S9633. doi: 10.4137/CMO.S9633 22619563 PMC3355865

[58] Roesch K, Hasenclever D, Scholz M. Modelling lymphoma therapy and outcome. Bulletin of mathematical biology. 2014;76(2):401–430. doi: 10.1007/s11538-013-9925-3 24338592 PMC3925304

[59] Bakhoum SF, Ngo B, Laughney AM, Cavallo JA, Murphy CJ, Ly P, et al. Chromosomal instability drives metastasis through a cytosolic DNA response. Nature. 2018;553(7689):467–472. doi: 10.1038/nature25432 29342134 PMC5785464

[60] Sun L, Wu J, Du F, Chen X, Chen ZJ. Cyclic GMP-AMP synthase is a cytosolic DNA sensor that activates the type I interferon pathway. Science (New York, NY). 2013;339(6121):786–791. doi: 10.1126/science.1232458 23258413 PMC3863629

[61] Lan YY, Londoño D, Bouley R, Rooney MS, Hacohen N. Dnase2a deficiency uncovers lysosomal clearance of damaged nuclear DNA via autophagy. Cell Reports. 2014;9(1):180–192. doi: 10.1016/j.celrep.2014.08.074 25284779 PMC4555847

[62] Mackenzie KJ, Carroll P, Martin CA, Murina O, Fluteau A, Simpson DJ, et al. cGAS surveillance of micronuclei links genome instability to innate immunity. Nature. 2017;548(7668):461–465. doi: 10.1038/nature23449 28738408 PMC5870830

[63] Ben-David U, Amon A. Context is everything: aneuploidy in cancer. Nature Reviews Genetics. 2020;21(1):44–62. doi: 10.1038/s41576-019-0171-x 31548659

[64] Cibulskis K, Lawrence MS, Carter SL, Sivachenko A, Jaffe D, Sougnez C, et al. Sensitive detection of somatic point mutations in impure and heterogeneous cancer samples. Nature Biotechnology. 2013;31(3):213–219. doi: 10.1038/nbt.2514 23396013 PMC3833702

[65] Storchova Z, Kuffer C. The consequences of tetraploidy and aneuploidy. Journal of Cell Science. 2008;121(23):3859–3866. doi: 10.1242/jcs.039537 19020304

[66] Shukla A, Nguyen THM, Moka SB, Ellis JJ, Grady JP, Oey H, et al. Chromosome arm aneuploidies shape tumour evolution and drug response. Nature Communications. 2020;11(1):449. doi: 10.1038/s41467-020-14286-0 31974379 PMC6978319

[67] S S, F K, N C, M A, Mj W, Kr C, et al. Clonal fitness inferred from time-series modelling of single-cell cancer genomes. Nature. 2021;595(7868):585–590. doi: 10.1038/s41586-021-03648-3 34163070 PMC8396073

[68] Nguyen B, Fong C, Luthra A, Smith SA, DiNatale RG, Nandakumar S, et al. Genomic characterization of metastatic patterns from prospective clinical sequencing of 25,000 patients. Cell. 2022;185(3):563–575.e11. doi: 10.1016/j.cell.2022.01.003 35120664 PMC9147702

[69] Abbosh C, Birkbak NJ, Wilson GA, Jamal-Hanjani M, Constantin T, Salari R, et al. Phylogenetic ctDNA analysis depicts early-stage lung cancer evolution. Nature. 2017;545(7655):446–451. doi: 10.1038/nature22364 28445469 PMC5812436

[70] Jamal-Hanjani M, Wilson GA, McGranahan N, Birkbak NJ, Watkins TBK, Veeriah S, et al. Tracking the Evolution of Non-Small-Cell Lung Cancer. The New England Journal of Medicine. 2017;376(22):2109–2121. doi: 10.1056/NEJMoa1616288 28445112

[71] Andor Noemi, Altrock Philipp, Jain Navami, Gomes Ana. Tipping cancer cells over the edge: the context-dependent cost of high ploidy. Cancer Research. 2021;. 10.1158/0008-5472.CAN-21-2794 PMC8898264 34785577

[72] Kaznatcheev A, Peacock J, Basanta D, Marusyk A, Scott JG. Fibroblasts and alectinib switch the evolutionary games played by non-small cell lung cancer. Nature Ecology & Evolution. 2019;3(3):450–456. doi: 10.1038/s41559-018-0768-z 30778184 PMC6467526

[73] Lynch AR, Arp NL, Zhou AS, Weaver BA, Burkard ME. Quantifying chromosomal instability from intratumoral karyotype diversity using agent-based modeling and Bayesian inference. eLife. 2022;11:e69799. doi: 10.7554/eLife.69799 35380536 PMC9054132

[74] Gao R, Bai S, Henderson YC, Lin Y, Schalck A, Yan Y, et al. Delineating copy number and clonal substructure in human tumors from single-cell transcriptomes. Nature Biotechnology. 2021;39(5):599–608. doi: 10.1038/s41587-020-00795-2 33462507 PMC8122019

[75] Sender R, Milo R. The distribution of cellular turnover in the human body. Nature Medicine. 2021;27(1):45–48. doi: 10.1038/s41591-020-01182-9 33432173

[76] Cheng J, Demeulemeester J, Wedge DC, Vollan HKM, Pitt JJ, Russnes HG, et al. Pan-cancer analysis of homozygous deletions in primary tumours uncovers rare tumour suppressors. Nature Communications. 2017;8(1):1221. doi: 10.1038/s41467-017-01355-0 29089486 PMC5663922

[77] Little P, Lin DY, Sun W. Associating somatic mutations to clinical outcomes: a pan-cancer study of survival time. Genome Medicine. 2019;11(1):37. doi: 10.1186/s13073-019-0643-9 31138328 PMC6540540

