# Supplementary material for: Intra-tumor heterogeneity, turnover rate and karyotype space shape susceptibility to missegregation-induced extinction
Source: PLoS Comput Biol. 2023 Jan 23;19(1):e1010815. doi: 10.1371/journal.pcbi.1010815 (PMC9917311; doi:10.1371/journal.pcbi.1010815)
Supplement: S1 Text — (PDF) [file pcbi.1010815.s001.pdf]

A general deterministic framework for modeling chromosome missegregations

Gregory J. Kimmel[+1], Richard J. Beck[+1], Xiaoqing Yu[2], Thomas Veith[1], Samuel Bakhoum[3], Philipp M. Altrock[4], Noemi Andor[*1]

[1]H. Lee Moffitt Cancer Center & Research Institute, Integrated Mathematical Oncology, Tampa, Florida.

[2]H. Lee Moffitt Cancer Center & Research Institute, Biostatistics & Bioinformatics, Tampa, Florida.

[3]Memorial Sloan Kettering Cancer Center, Human Oncology & Pathogenesis Program, New York City, New York.

[4]Max Planck Institute for Evolutionary Biology, Department for Evolutionary Theory, Plön, Germany

[+]Authors contributed equally to this work

[*]Corresponding author: noemi.andor@gmail.com

February 4, 2023

Supplementary Information

Key terms and definitions

- A chromosome **mis-segregation** segregates chromosomes asymmetrically among daughter cells, causing an aneuploid state.
- **Aneuploidy** := a chromosome number that is not a multiple of the haploid complement. An **euploid** cell carries a multiple of the same set of chromosomes. Haploids (1 copy of each chromosome), diploids (2 copies) and polyploids (> 2 copies) are all examples of euploidy.
- **Karyotype** := a numeric vector with one entry per chromosome **type** (e.g. we have 22 types when considering the set of human autosomes). Each entry indicates the **copy number** of that type of chromosome. Each karyotype corresponds to a compartment of a multi-compartment ODE.
- **Ploidy** := the total number of chromosomes occurring in the nucleus of a cell. Also referred to as **DNA content** in the continuous setting.
- **Karyotype composition** := the relative frequency of all karyotypes in a population at a given time.
- **Set of viable karyotypes** := all possible karyotypes associated with non-negative growth rates. This can either be a single **interval** (e.g. 22 - 88 chromosomes) or multiple intervals of karyotype viability interspersed between nonviable regions.

1.1 Relationship between timescale and characteristic amount of DNA shifted per missegregation

Let T_p be the time it takes for a mitotic cell to segregate its chromosomes among daughter cells. Given that cells divide at a rate λ and that only dividing cells missegregate (at a rate β), we expect the timescale of missegregations to be proportional to $T_p\lambda\beta$. We approximate the shift in DNA content over time as Fickian diffusion, which gives us L_p, the characteristic amount of DNA content shifted during a missegregation event, as: $L_p \sim \sqrt{T_p\lambda\beta}$ [1]. From here we can approximate the timescale on which missegregations happen as: $T_p \sim L_p^2/(\beta\lambda)$.

1.2 Numerical methods for calculating the critical curve

Critical curves are derived by leveraging the fact that the critical turnover rate for a given β is when the rate of missegregation induced cell death equals the growth rate $(\lambda - \mu)$. Let J be the Jacobian defined in (10). The system reaches a steady state when $n_i J = 0$. Nontrivial solutions can be found by choosing functions for the mis-segregation rate β and death rate μ (which parameterise the matrix J) such that the dominant eigenvalue is zero. A robust method for determining pairs of values (B, M) on the critical curve (which can cope with heterogeneous functions for β, μ), is to: 1) choose an input value B for the function $\beta(i, B)$, then 2) find the value of M parameterizing $\mu(i, M)$ such that the dominant eigenvalue of J is equal to zero. The eigenvector corresponding to this eigenvalue is the steady state of the system. Although this method is robust, it requires multiple generations of the matrix J with different values of M and so can become slow when J is a large matrix. A considerable

time saving can be achieved when death rate does not depend on copy number state, i.e. $\mu = M$. In that case, J can be expressed in terms of the adjusted matrix J^*:

$$J = J^* + \mu I, \tag{16}$$

where J^* represents the transition matrix with $\mu = 0$ and I is the identity matrix. It can be shown that the eigenvectors of J are identical to those of J^*, therefore the steady state population distribution (i.e. the karyotype composition) does not depend on the death rate μ and is given by the dominant eigenvector of J^*. Denoting this eigenvector $\bar{n_i}$, after normalising $\bar{n_i}$ such that the entries sum to one, $\sum_i \bar{n_i} J^*$ gives the net growth rate of the cell population when $\mu = 0$ and the population *distribution* has reached steady state (although net population growth still occurs when $\mu = 0$). Therefore, the true steady state (and therefore the critical curve) is found by choosing the value of μ which exactly negates this growth, i.e: $\mu = \sum_i \bar{n_i} J^*$. We note that if death rates are heterogeneous then J^* is no longer independent of the death rate μ, i.e. the approach relies on the assumption of homogeneous rates.

Both of the above approaches work when a single chromosome type has a finite viable copy number interval as well as when multiple chromosomes have finite intervals (i.e. n_i becomes $n_{\vec{i}}$). However both become infeasible when we have $k \gg 1$ chromosome types, each with multiple copies, as the matrix J becomes too large. However the critical curve can still be generated by extrapolating from the single chromosome case. Summing over the rows of the matrix q_{ij} (eq. (7)) gives a probability vector q_i^*, which contains the probability that a daughter of a cell with i chromosome copies receives a viable number of copies. The overall probability that a daughter cell from the steady state population receives an unviable number of copies for a single chromosome type is therefore: $q_d = 1 - q_i^* \cdot \bar{n_i}$. The probability that a daughter receives viable copy numbers for all k chromosome types is: $q_a^k = (1 - q_d)^k = (q_i^* \cdot \bar{n_i})^k$. The rate of change of the alive cell population is given by:

$$\frac{dn_a}{dt} = \lambda n_a (2q_a^k - 1) - \mu n_a, \tag{17}$$

,

where n_a is the total number of alive cells. Setting to zero, we have $\mu = 2q_a - 1$.

1.3 MIE is impossible for infinite compartments - continuum argument

To appeal to the continuum model we have to assume that only small changes in DNA content (small jumps) happen during a given cell division. Under this assumption equation (9) can be expanded as:

$$\frac{dn_i}{dt} = \lambda_{i+1}\beta_{i+1}n_{i+1} - 2\lambda_i\beta_i n_i + \lambda_{i-1}\beta_{i-1}n_{i-1} + (\lambda_i - \mu_i)n_i. \tag{18}$$

Defining $p = i/K$, and $\Delta p = 1/K$, for $K \to \infty$ we have:

$$\lambda_{i+1}\beta_{i+1}n_{i+1} - 2\lambda_i\beta_i n_i + \lambda_{i-1}\beta_{i-1}n_{i-1} \to \frac{1}{K^2}\frac{\partial^2}{\partial p^2}(\beta\lambda n). \tag{19}$$

This leads to:

$$\frac{\partial n}{\partial t} = (\lambda - \mu)n + \frac{1}{K^2}\frac{\partial^2}{\partial p^2}(\beta\lambda n), \tag{20}$$

where $p \in (0,1)$ and $n(0) = n(1) = 0$. We make use of the Ansatz, $n = e^{\alpha t}U(p)$, which results in the eigenvalue problem:

$$\alpha U = \frac{1}{K^2}(\beta\lambda U)'' + (\lambda - \mu)U. \tag{21}$$

For simplicity, we define $q = \frac{1}{K^2}\beta\lambda$ and convert to Sturm-Liouville form:

$$\begin{aligned}\alpha U &= qU'' + 2q'U' + (\lambda - \mu + q'')U \\ \alpha q U &= (q^2U')' + q(\lambda - \mu + q'')U.\end{aligned}$$

Multiplying by U and integrating over p, we obtain an equation for α (the Rayleigh Quotient):

$$\alpha = \frac{\int_0^1 -q^2(U')^2 + q(\lambda - \mu + q'')U^2 \, dp}{\int_0^1 qU^2 \, dp} = \frac{\int_0^1 -\frac{(\beta\lambda)^2}{K^2}(U')^2 + \beta\lambda\left[\lambda - \mu + \frac{(\beta\lambda)''}{K^2}\right]U^2 \, dp}{\int_0^1 \beta\lambda U^2 \, dp}. \tag{22}$$

For $K \to \infty$ we may neglect the terms with K^{-2} and obtain

$$\alpha = \frac{\int_0^1 \beta\lambda(\lambda - \mu)U^2 \, dp}{\int_0^1 \beta\lambda U^2 \, dp}. \tag{23}$$

Provided that $r = \lambda - \mu > 0$ for all p we define $m = \inf(\beta\lambda(\lambda - \mu))$ and $P = \sup(\beta\lambda)$, it follows that

$$\alpha \geq \frac{m}{P} > 0.$$

Finally, we note that the Rayleigh quotient satisfies $\alpha_{\min} \leq \alpha \leq \alpha_{\max}$ for all amendable test functions U, where $\alpha_{\min}, \alpha_{\max}$ are the smallest and largest eigenvalues, respectively. Hence, $\alpha_{\max} > 0$ and so MIE is impossible.

Note that the proof did not rely on the whole integral having $r > 0$. Suppose that we have an almost everywhere differentiable function U that is 0 on $[0,1]\backslash E$, with $E \subset [0,1]$ and m, P defined as above. Then (23) becomes:

$$\alpha = \frac{\int_E \beta\lambda(\lambda - \mu)U^2 \, dp}{\int_E \beta\lambda U^2 \, dp} \geq \frac{m}{P} > 0, \tag{24}$$

provided that $|E| > 0$.

On a final note, this may seem to imply that we have shown that a *finite* number of compartments in the small jump case can't have MIE, but this would be false. We must take a closer look at the passage to equilibrium. For simplicity suppose the viable karyotype interval is at the beginning $i = 1, \ldots, f(K)$ where $r > 0$. Defining $p = i/K$ as before, note that the viable interval length is on the order of $f(K)/K$. For the measure of this interval to remain finite, we require that $f(K) \to cK$ for $K \to \infty$ for some $0 < c < 1$ to rule out MIE. The viable interval length must grow at the same order as the number of viable intervals grows, in order to be able to rule out MIE using this method.

1.4 Ruling out MIE

Here, we establish sufficient conditions for MIE to not occur. These are based on Gershgorin's circle (GC) theorem which states that for matrix A with elements a_{ij}, the eigenvalues ψ satisfy:

$$|\psi - a_{ii}| \leq \sum_{j \neq i} |a_{ij}| = R_i. \tag{25}$$

Put differently, every eigenvalue of A lies within at least one of the discs with center a_{ii} and radius R_i.

Since MIE can be evaded if the maximum eigenvalue exceeds 0, a sufficient condition is that none of Gershgorin's circle contain a part of the negative reals. That is $a_{ii} - R_i > 0$ for all i.

Using GC, a sufficient condition to avoid MIE is given by:

$$\beta < \beta_c = \frac{1}{4}\left(1 - \frac{\mu}{\lambda}\right). \tag{26}$$

Of course, we know that for large K, *no* missegregation rate will lead to MIE; this just provides a sufficient condition.

If λ, β and μ vary with ploidy i, we can still obtain a sufficient condition to avoid MIE given by:

$$\lambda_i(1 - 3\beta_i) - \mu_i - \lambda_{i+1}\beta_{i+1} > 0, \text{ for all } i. \tag{27}$$

If β is homogeneous, then this reduces to

$$\beta < \beta_c = \frac{1}{3 + \lambda_{i+1}/\lambda_i}\left(1 - \frac{\mu_i}{\lambda_i}\right), \text{ for all } i. \tag{28}$$

The general problem (i.e. any arbitrary relation between λ, β and μ) can be handled analogously, but conclusions can only be made for specific forms of $\mathbf{q}$. We require both (11)-(12). As both of these need to be positive, we can find the minimum of these, which will provide sufficient condition to escape MIE.

Let us consider a more complicated example to show the power of this method. Suppose that λ, μ are homogeneous, but that $q_{ij} = q(i \to j)$ obeys the following relationship

$$q(i \to i \pm h) = \kappa_i \beta^h, \; h = 0, 1, \ldots, i. \tag{29}$$

This $\mathbf{q}$ can be thought of as one that favors small jumps but all jumps are permitted. Let us suppose that there is no limit on the number of compartments, then:

$$1 = \sum_{h=0}^{i} q(i \to i + h) = \kappa_i \sum_{h=0}^{i} \beta^h = \frac{\kappa_i(1 - \beta^{i+1})}{1 - \beta}.$$

This implies the relation for κ_i:

$$\kappa_i = \frac{1 - \beta}{1 - \beta^{i+1}}. \tag{30}$$

We now look at the sum in (11) (note the symmetry $q(i \to i+h) = q(i \to i-h)$),

$$2\kappa_i \sum_{h=1}^{i} \beta^h = \frac{2\beta(1-\beta^i)}{1-\beta^{i+1}} \tag{31}$$

The sum in (12) is trickier since in this case we are looking at the possibilities $q(i+h \to i)$ and it is clear that $q(i+h \to i) \neq q(i-h \to i)$ (because any given ploidy state i is more likely to emerge from missegregations in a higher ploidy cell than in a lower ploidy cell). We look at the first part of the sum first

$$\sum_{h=1}^{\infty} q(i+h \to i) = \sum_{h=1}^{\infty} \kappa_{i+h}\beta^h = (1-\beta)\sum_{h=1}^{\infty} \frac{\beta^h}{1-\beta^{i+h+1}} = \beta + \mathcal{O}(\beta^{3+i}),$$

valid when $\beta \ll 1$. The sum from the other direction is nonzero only if $h \geq \lceil i/2 \rceil$:

$$\sum_{h=\lceil i/2 \rceil}^{i-1} q(i-h \to i) = \sum_{h=\lceil i/2 \rceil}^{i-1} \kappa_{i-h}\beta^h = (1-\beta)\sum_{h=\lceil i/2 \rceil}^{i-1} \frac{\beta^h}{1-\beta^{i-h+1}} = \beta^{\lceil i/2 \rceil} - \beta^i + \mathcal{O}(\beta^{i+1}).$$

Therefore, the sum term in (12) ($\sum_{j\neq k} \lambda_j q_{jk}$) becomes:

$$\beta + \beta^{\lceil i/2 \rceil} - \beta^i + \mathcal{O}(\beta^{1+i}). \tag{32}$$

The bigger sum sets the threshold and is maximal for large i. With this choice of $\mathbf{q}$, it is (11) which is the dominant condition. Thus, a sufficient condition to escape MIE is given by:

$$\beta < \beta_c = \frac{1}{4}\left(1 - \frac{\mu}{\lambda}\right). \tag{33}$$

The appearance of this relation potentially suggests a fundamental limit on a kernel of this type. Indeed, it is not hard to show that for general $\mathbf{q}$ with $q_{ii} = 1 - \beta_i$, (11) yields

$$\beta_i < \frac{1}{4}\left(1 - \frac{\mu_i}{\lambda_i}\right), \text{ for all } i.$$

Since both (11) and (12) must be met, the above condition is the upper bound on the β_i's required to evade MIE.

1.5 Extensions to model whole-genome doubling and micronuclei formation

Though WGDs are technically feasible in the model (through an i parent giving birth to $2i$ and 0 ploidy offspring), this ignores the biological mechanisms for how WGD events may occur. There are two mechanisms for WGD [2,3] – endoreplication (i.e. completely skipping mitosis) and cytokinesis failure which often generates binucleated whole genome doubled cells. Chromosomes in the two nuclei can then merge the subsequent mitosis after nuclear envelope breakdown. We can modify equation (9) to account for WGD by introducing terms

$$\frac{dn_i}{dt} = \sum_j \lambda_j n_j q_{ji} - \lambda_i n_i(1-q_{ii}) - \mu_i n_i - \theta_i n_i + \theta_{i/2} n_{i/2},$$

where $\theta_{i/2} = 0$ if $i/2$ is not an integer.

Mounting evidence suggests that a cell which mis-segregates many chromosomes has a higher risk to die than a cell mis-segregating only one chromosome [4, 5]. Our current model structure however does not distinguish between these scenarios. To account for this intolerance, we can amend the inflow term by introducing a karyotype transition coefficient ϕ_{ij}. One possible form $\phi = (1+|i-j|)^\alpha$ with $\alpha < 0$, where only relative distance is important. Other forms that take the parental state into account could also be considered. Combining this with the above, leads to

$$\frac{dn_i}{dt} = \sum_j \phi_{ji}\lambda_j n_j q_{ji} - \lambda_i n_i(1 - q_{ii}) - \mu_i n_i - \theta_i n_i + \theta_{i/2} n_{i/2}.$$

The only requirement is that ϕ_{ij} must fall off with increasing distance between states.

Yet another assumption, which could be eventually relaxed is the ploidy conservation in equation (3). The errors during mitosis could lead to the formation of micronuclei which are not directly involved in further downwind mitosis [6–10]. This amounts to replacing equation (3) with $2i_k \geq j_k^{(1)} + j_k^{(2)}$ for all k.

Table A: ***In vivo* tumor dynamics across nine cancer types**. Birth rates taken from [11] and growth rates taken from multiple sources (cited in third column). The death rate is inferred. Many of the birth rates are comparable, but the net growth rates vary over orders of magnitude, which implies very different death rates. Last column shows percent contribution of respective normal human cell types to a total daily mass turnover of 80 g (taken from [12]) – these are correlated to both birth and death rates reported in tumors (Pearson $r \geq 0.923$; $P < 0.026$).

Cancer type	birth rate (λ) [11]	growth rate (r)	death rate ($\mu = \lambda - r$)	normal turnover (%)
Head/Neck	0.192 (0.015 - 1.667)	0.007 (0.003 - 0.033) [13]	0.185 (0 - 1.664)	NA
Esophageal	0.207 (0.018 - 0.625)	0.154 (0.035 - 0.347) [14]	0.053 (0 - 0.59)	NA
Colorectal	0.239 (0.019 - 1.111)	0.003 (0.0003 - 0.038) [15]	0.236 (0 - 1.111)	41 [12]
Rectal	0.303 (0.179 - 0.417)	0.005 (0.0004 - 0.013) [16]	0.298 (0.166 - 0.416)	41 [12]
Breast	0.122 (0.021 - 0.556)	0.003 (0.0003 - 0.016) [17]	0.119 (0.005 - 0.555)	NA
Cervix	0.25 (0.159 - 0.323)	0.002 (0.0004 - 0.012) [18]	0.248 (0.147 - 0.322)	NA
Melanoma	0.139 (0.024 - 0.286)	0.007 (0.002 - 0.014) [19]	0.132 (0.01 - 0.284)	4 [12]
SC Lung	0.122 (0.036 - 0.667)	0.008 (0.005 - 0.013) [20]	0.114 (0.023 - 0.662)	0.5 [12]
D-LBCL	0.063 (0.043 - 0.4)	0.023 (0.01 - 0.5) [21]	0.04 (0 - 0.39)	1.7 [12]

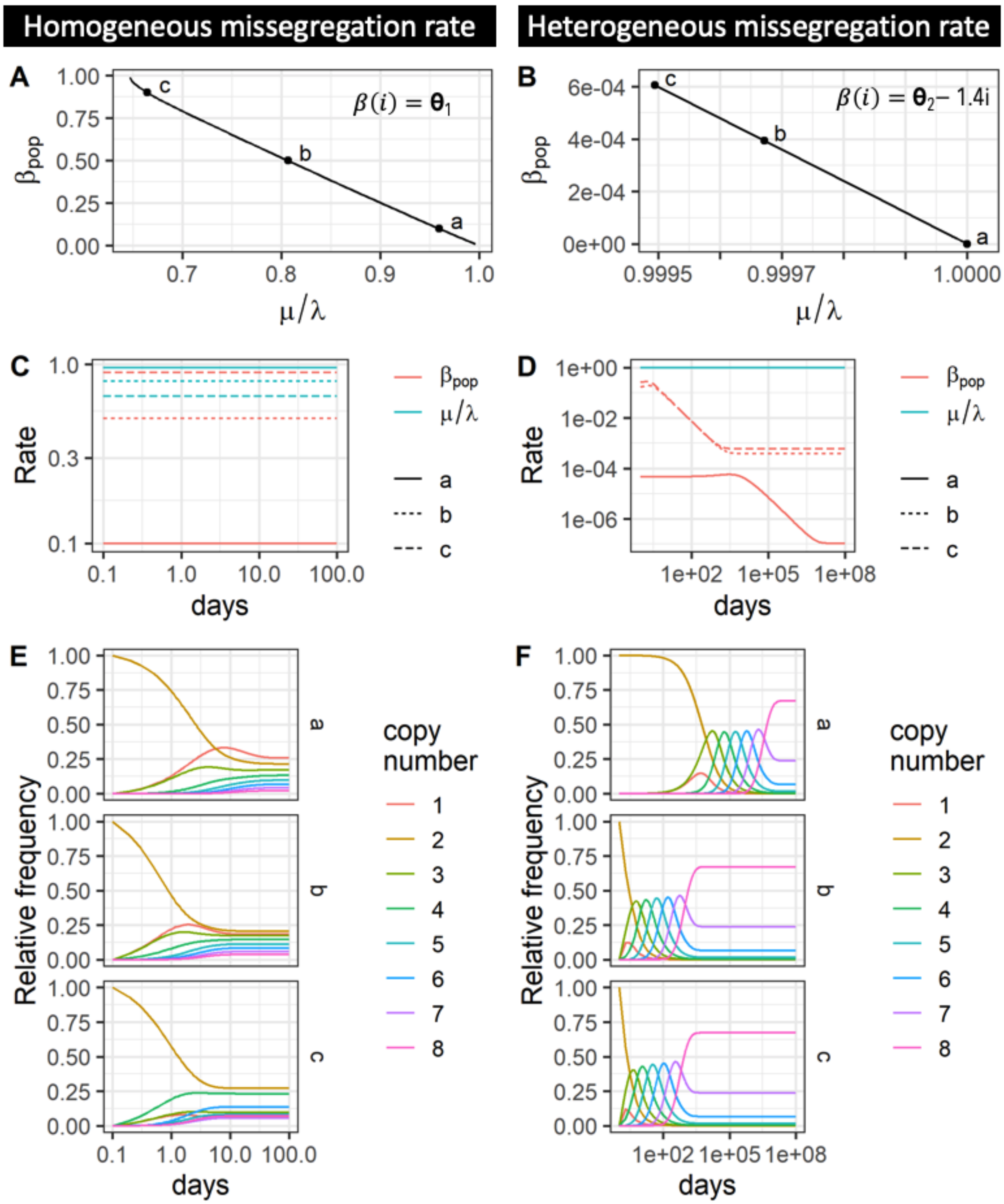

Fig A: **Predicted changes in missegregation rate during tumor evolution.** (A-B) Critical curves displayed as a function of the population average missegregation rate (y-axis) and departure from homeostasis (1 - turnover rate; x-axis). $\lambda := 1$ in all calculations, i.e. cells divide once per day. Copy number ranges from $[1, 8]$ and death rate is independent of copy number ($\mu(i) := M$), whereas missegregation is either also independent (A, $\beta(i) := \Theta_1$) or dependent on copy number (B, $\beta(i) := \Theta_2 - 1.4i$). (C-D) Population average missegregation and turnover rates over time are shown for three parameter combinations $\theta \in a, b, c$ highlighted in (A,B) respectively. (E-F) Evolution of karyotype composition for each of the three parameter combinations is also shown.

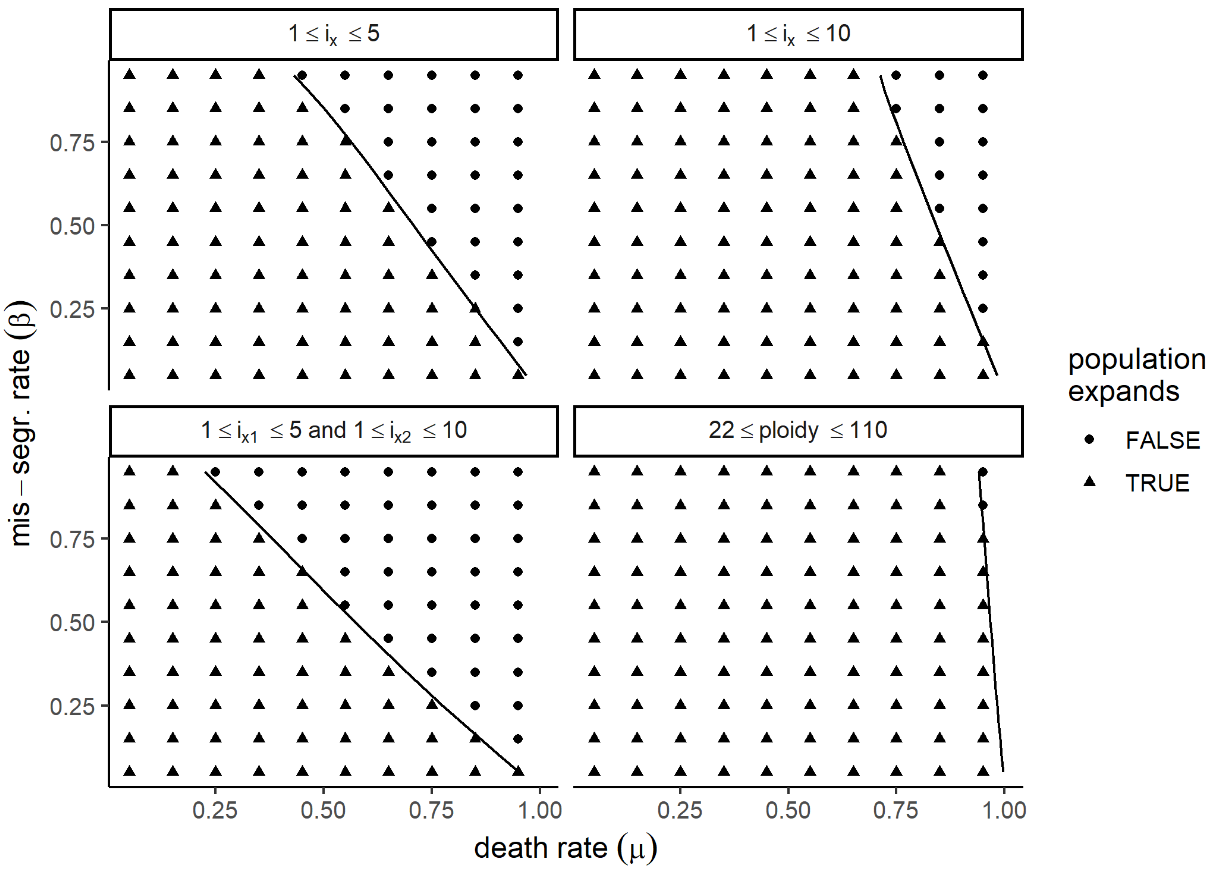

Fig B: **Numerical simulations confirm theoretical MIE curves**. Numerical simulations confirm that the theoretical critical curves shown in Fig 2A,B separate exponential growth from population extinction.

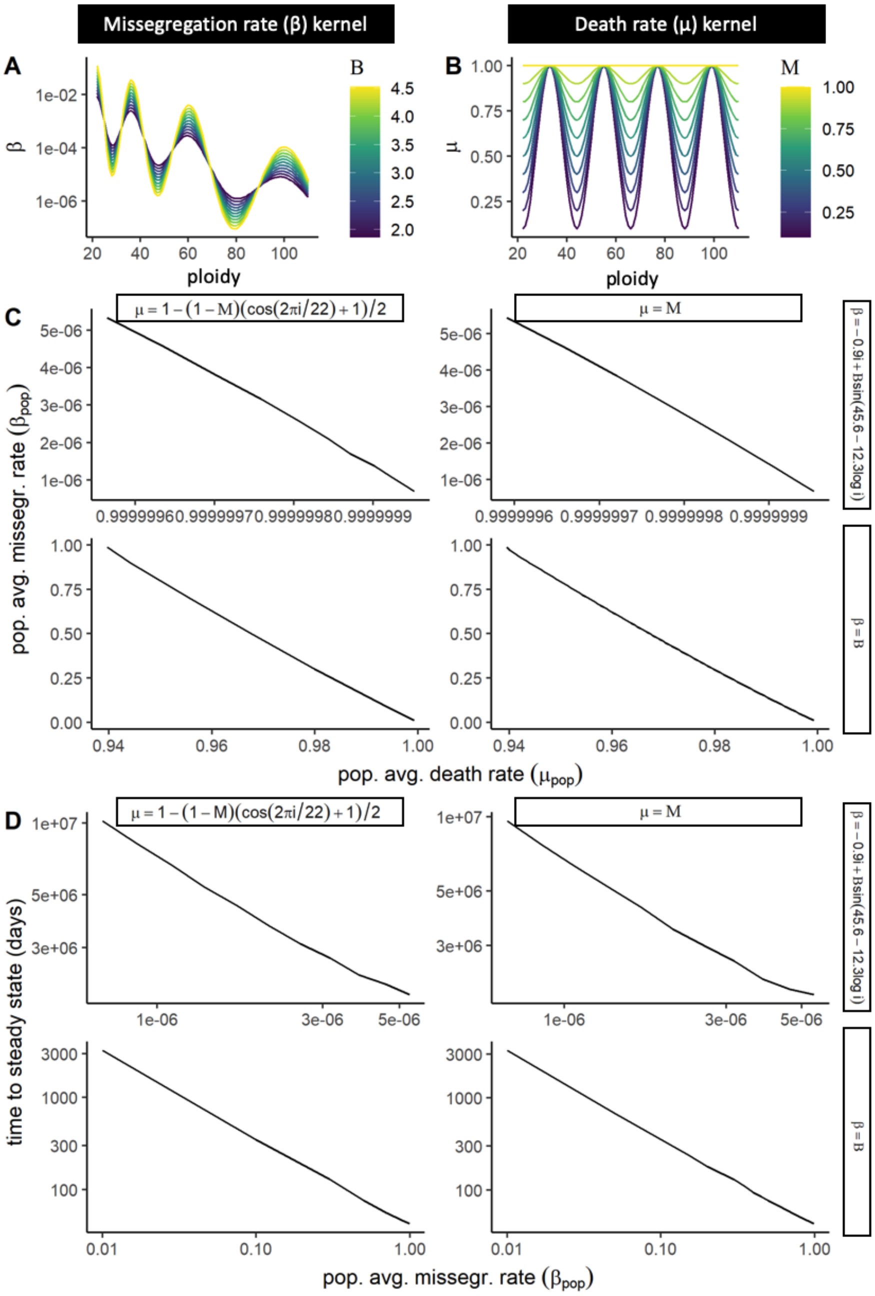

Fig C: **Heterogeneous missegregation and death rates can render MIE impossible**. Kernels used to model intra-tumor heterogeneity in missegregation- (A) and turnover rates (B). (C) Critical curves calculated assuming homogeneous or heterogeneous missegregation and turnover rates are compared with each other. Functions used to model heterogeneous rates are displayed as row and column labels and have ploidy (i) as parameter. For each of these functions, we calculated the critical missegregation rate parameter (B) and death rate parameter (M). Note that for $M = 1$, the turnover rate function in the first column becomes the constant rate from the second column. MIE requires $M \to 1$, explaining why critical curves look identical in both columns. (D) We also simulated the ODE assuming combinations of the functional forms of missegregation and turnover rates shown in C. Shown here is the number of days needed to reach steady state karyotype composition as a function of population average missegregation rate.

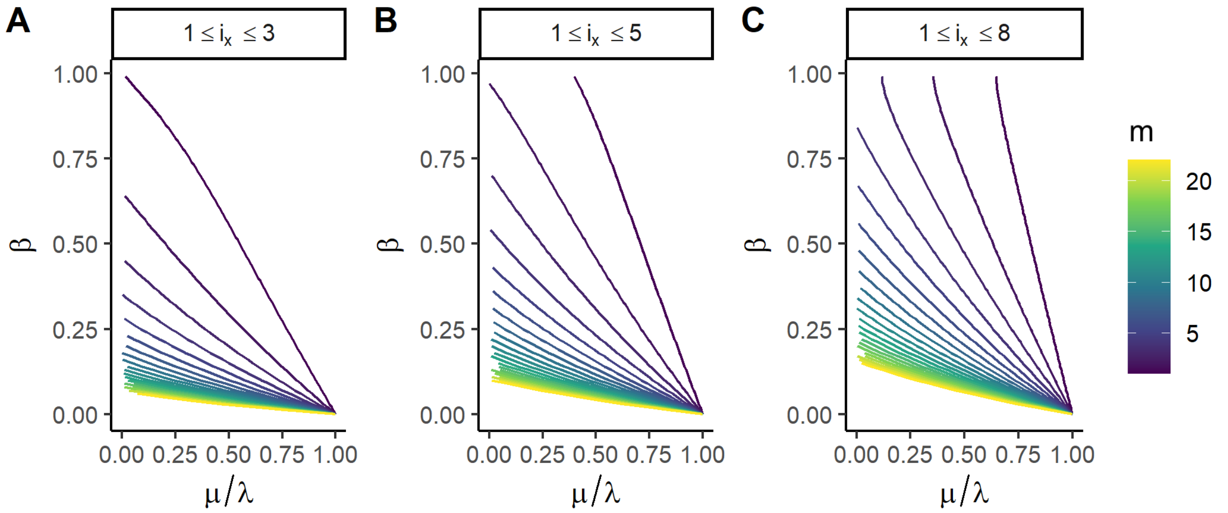

Fig D: **Risk of MIE as a function of the number of chromosome types with finite viable copy number intervals**. (A-C) Critical curves were obtained by finding $(\beta, \frac{\mu}{\lambda})$ for which the maximum eigenvalues of the Jacobian (eq. (10)) is 0. We assume existence of up to $m = 22$ critical chromosomes, where intervals of viable karyotypes for each chromosome type $x \in 1..m$ are defined by $k_x \leq i_x \leq K_x$. We calculate the critical curves assuming cell viability is restricted by all 22 of the chromosomes or by only a subset of them (color code). Hereby we assume each chromosome must have at least one and no more than three (A), five (B) or eight copies respectively (C).

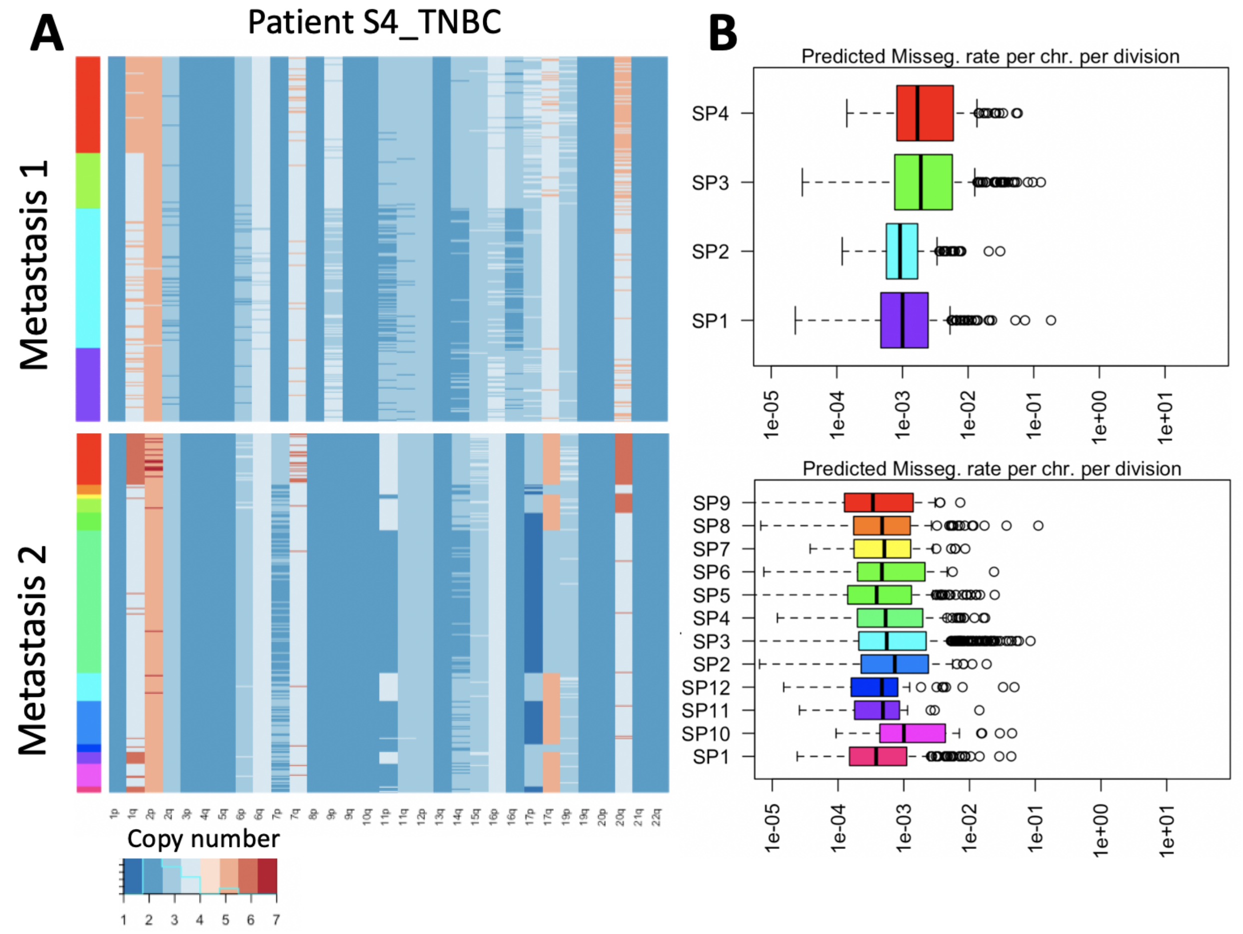

Fig E: **Karyotype profiles inferred for two metastatic TNBC samples** . (A) Copy number profiles from two of the metastatic samples from patient *S4_TNBC1* (marked red in Fig 3B,D) were clustered into subpopulations of cells with unique karyotypes (left color code). The two metastatic samples originate from the same patient and have significant similarities in their karyotype profiles, even though they were called independently from each other. (B) Inferred mis-segregation rates per cell division per chromosome for each subpopulation in (A).

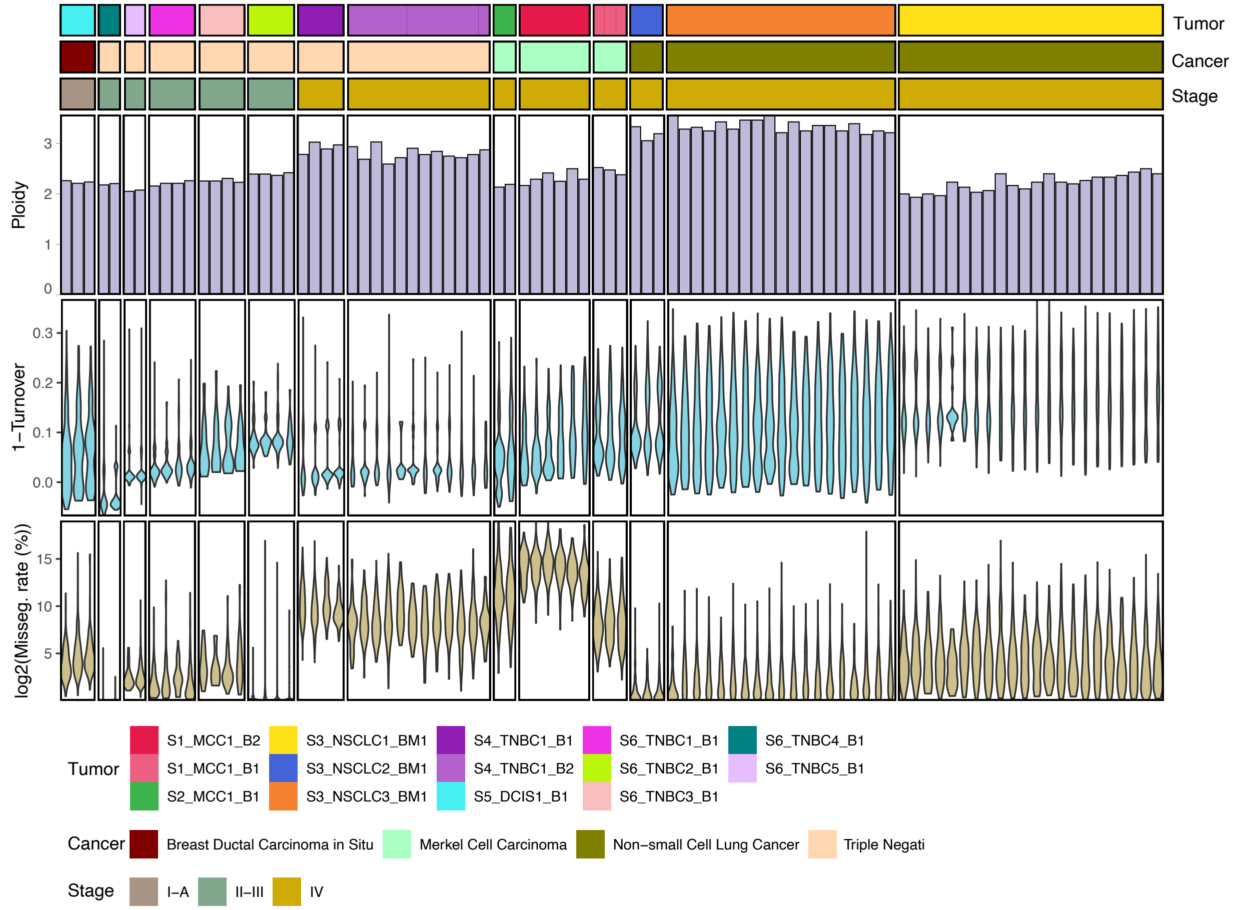

Fig F: **Variability in turnover, missegregation and ploidy across coexisting subpopulations in 14 tumors**. Every column represents a subpopulation of cells with unique karyotype. Subpopulations from the same tumor are grouped together. Their ploidy (first row), departure from homeostasis (second row) and log2-transformed % missegregation rate (third row) are shown.

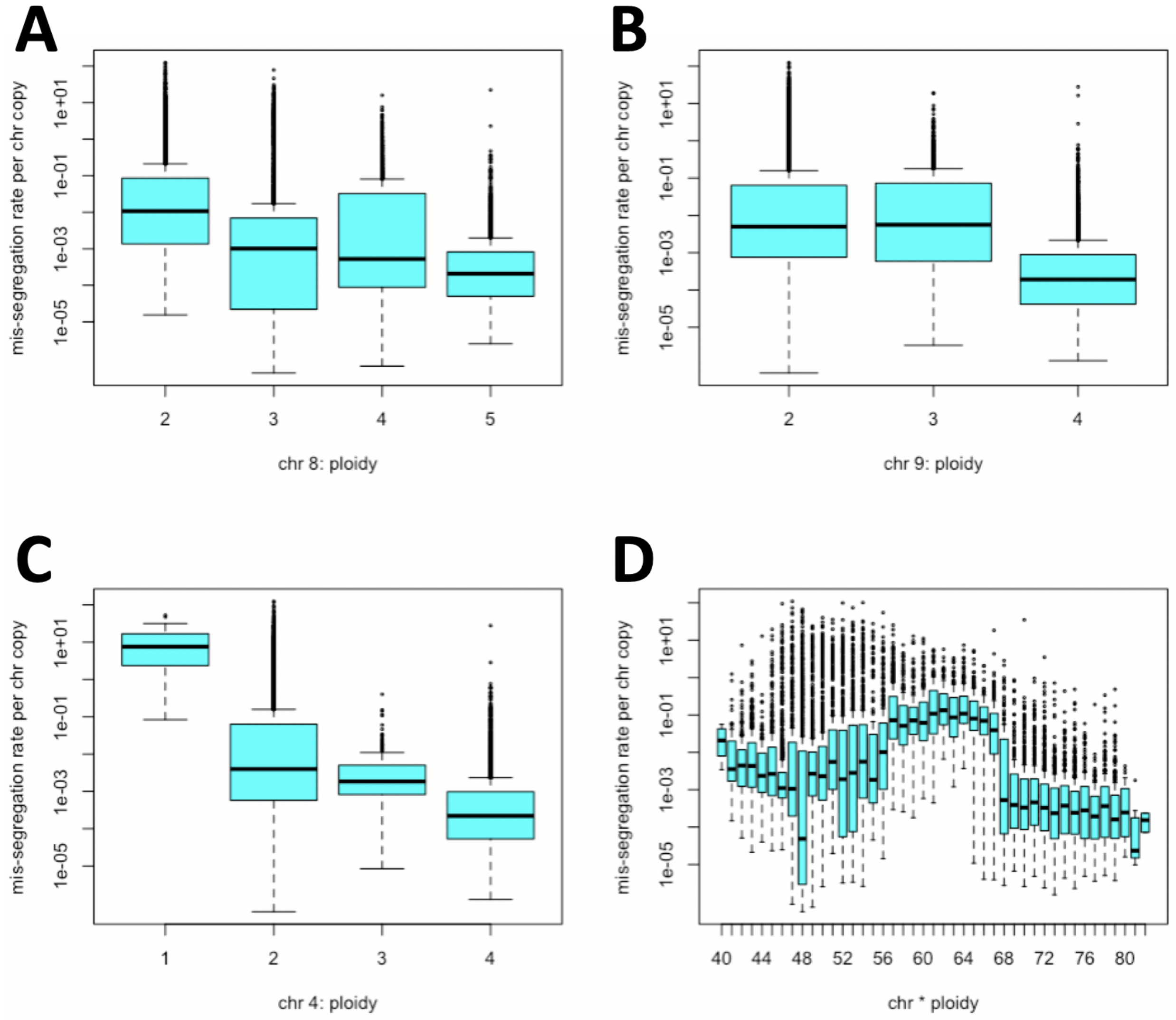

Fig G: **Relationship between karyotype and missegregation rates**. Mis-segregation rates per chromosome (y-axis) vary with the copy number (x-axis) of specific chromosomes (A-C) and with ploidy (i.e. copy numbers of all chromosomes in aggregate; D).

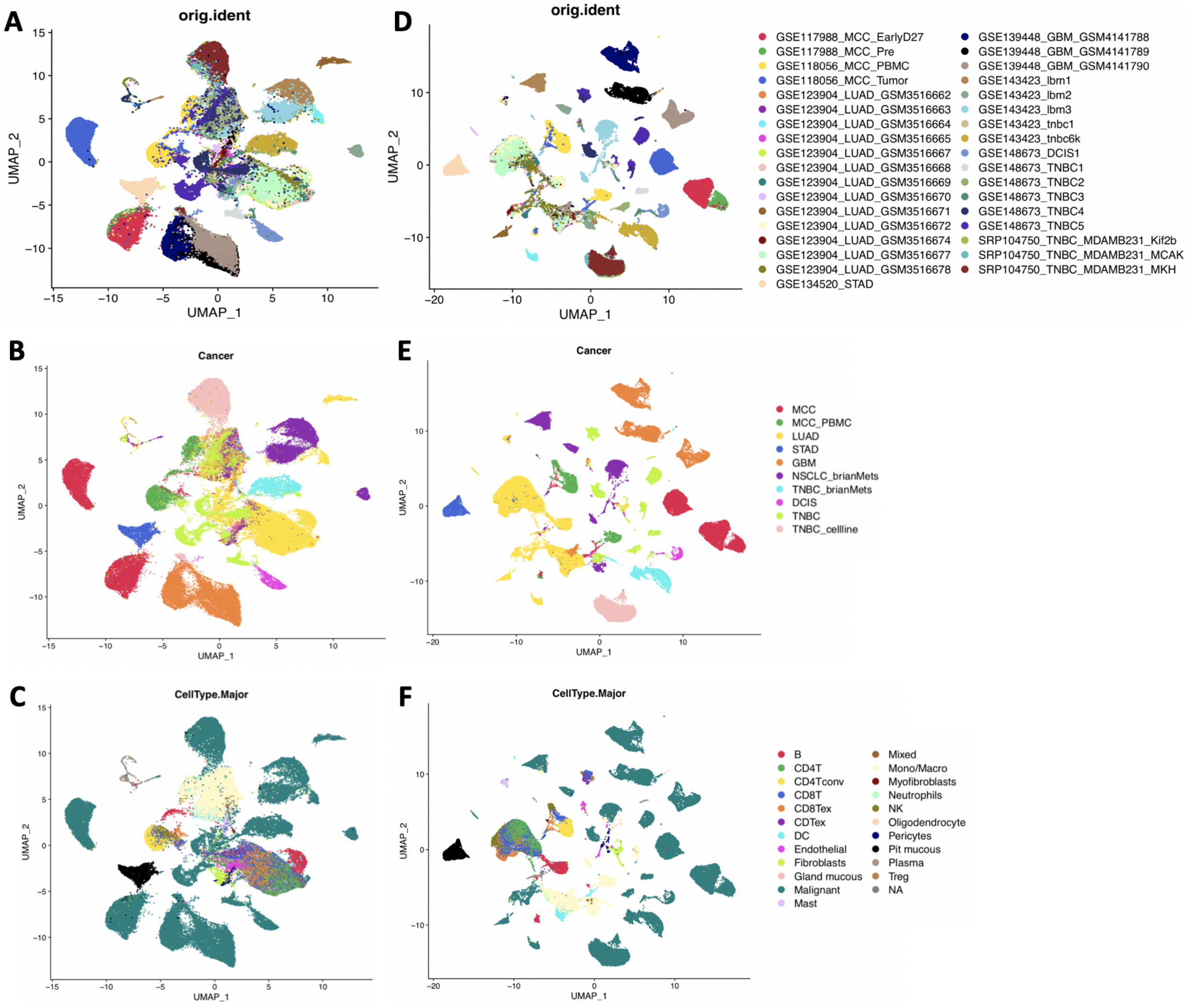

Fig H: **Effect of normalization and scaling for integration of scRNA-seq datasets.** (A-C) UMAP plots using the normalized and scaled data, with the cells labeled by (A) sample name, (B) cancer type of the patients and (C) cell types. Tumor cells cluster by sample origin, and tumor cells from similar cancer types are closer to each other. The PBMC sample of the MCC patient clusters with other normal cells, instead of with the tumor cells from the same patient. Normal cells cluster mostly by cell type. (D-F) Analogous plots to (A-C), but generated using non-normalized data. Generally the cells are more clustered by study, and the tumor cells from different samples are less distinguished, emphasizing the need for normalization and scaling.

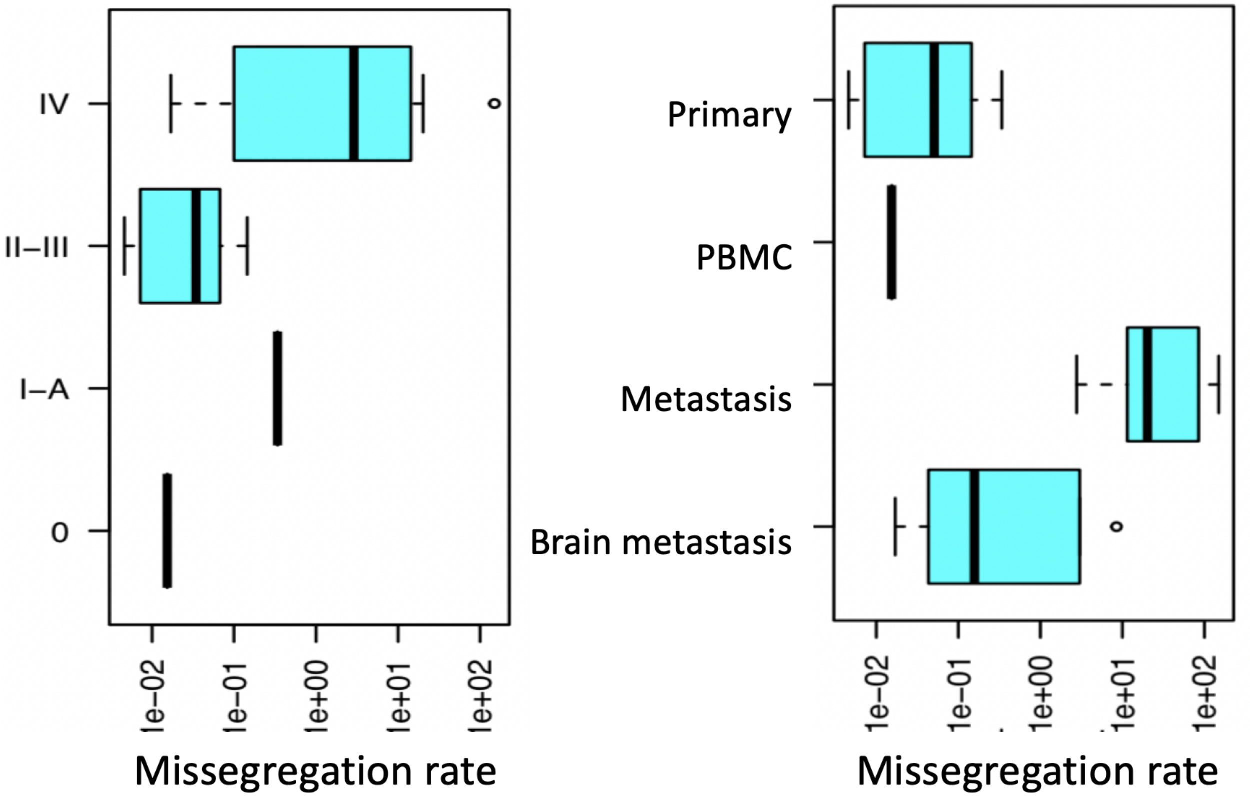

Fig I: **Mis-segregation rates inferred from scRNA-seq derived gene signatures**. Distribution of median mis-segregation rate across all sequenced G1 cells of a given sample are grouped by disease stage (left) or site (right). PBMC:= peripheral blood mononuclear cells.

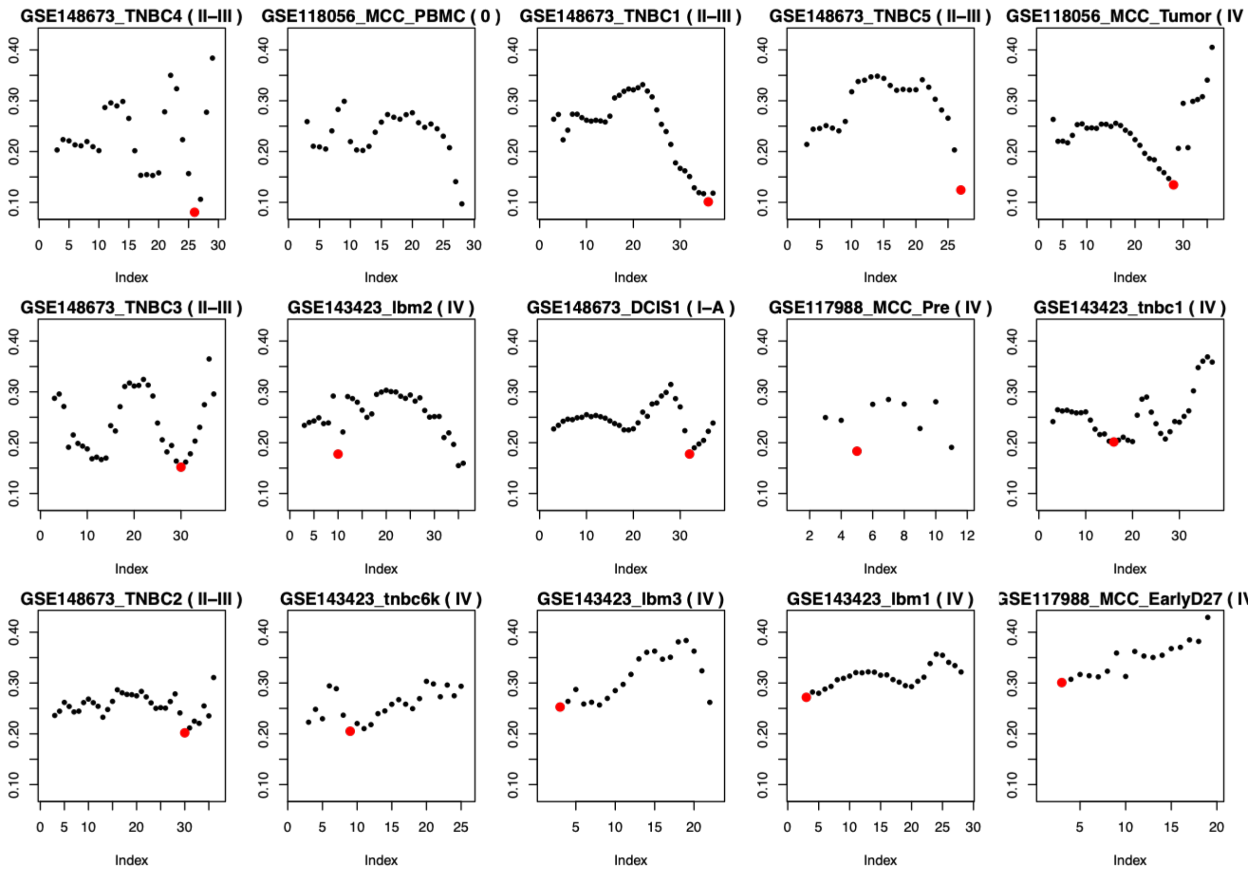

Fig J: **Classification of aneuploid chromosomes with scRNA-Seq derived bias profiles**. Minimum deviation from integer copy numbers (y-axis) guides the choice of x (highlighted red). The more to the right the minimum, the more chromosomes are assumed to be diploid. Shown are the bias profiles for 15 scRNA-seq samples, sorted by global minimum. Samples from late-stage tumors (stage IV) have by trend more non-diploid chromosome arms and their global minimum is higher. Range of x-axis varies because only chromosomes that express a sufficient number of genes in both tumor and normal cells were considered.

References

1. Cantor B. Fick's Laws: Diffusion. In: Cantor B, editor. The Equations of Materials. Oxford University Press; 2020. p. 0. Available from: https://doi.org/10.1093/oso/9780198851875.003.0007.

2. Ganem NJ, Storchova Z, Pellman D. Tetraploidy, aneuploidy and cancer. Current opinion in genetics & development. 2007;17(2):157–162. Publisher: Elsevier.

3. Shi Q, King RW. Chromosome nondisjunction yields tetraploid rather than aneuploid cells in human cell lines. Nature. 2005 Oct;437(7061):1038–1042.

4. Thompson SL, Compton DA. Proliferation of aneuploid human cells is limited by a p53-dependent mechanism. The Journal of Cell Biology. 2010 Feb;188(3):369–381.

5. Santaguida S, Richardson A, Iyer DR, M'Saad O, Zasadil L, Knouse KA, et al. Chromosome mis-segregation generates cell cycle-arrested cells with complex karyotypes that are eliminated by the immune system. Developmental cell. 2017 Jun;41(6):638–651.e5. Available from: https://www.ncbi.nlm.nih.gov/pmc/articles/PMC5536848/.

6. Crasta K, Ganem NJ, Dagher R, Lantermann AB, Ivanova EV, Pan Y, et al. DNA breaks and chromosome pulverization from errors in mitosis. Nature. 2012;482(7383):53–58. Publisher: Nature Publishing Group.

7. Zhang CZ, Spektor A, Cornils H, Francis JM, Jackson EK, Liu S, et al. Chromothripsis from DNA damage in micronuclei. Nature. 2015;522(7555):179–184. Publisher: Nature Publishing Group.

8. Hatch EM, Fischer AH, Deerinck TJ, Hetzer MW. Catastrophic nuclear envelope collapse in cancer cell micronuclei. Cell. 2013;154(1):47–60. Publisher: Elsevier.

9. Leopardi P, Marcon F, Dobrowolny G, Zijno A, Crebelli R. Influence of donor age on vinblastine-induced chromosome malsegregation in cultured peripheral lymphocytes. Mutagenesis. 2002 Jan;17(1):83–88.

10. Bakhoum SF, Ngo B, Laughney AM, Cavallo JA, Murphy CJ, Ly P, et al. Chromosomal instability drives metastasis through a cytosolic DNA response. Nature. 2018 Jan;553(7689):467–472. Available from: https://www.nature.com/articles/nature25432.

11. Rew D, Wilson G. Cell production rates in human tissues and tumours and their significance. Part II: clinical data. European Journal of Surgical Oncology (EJSO). 2000;26(4):405–417. Publisher: Elsevier.

12. Sender R, Milo R. The distribution of cellular turnover in the human body. Nature Medicine. 2021 Jan;27(1):45–48. Number: 1 Publisher: Nature Publishing Group. Available from: https://www.nature.com/articles/s41591-020-01182-9.

13. Forster JC, Douglass MJ, Harriss-Phillips WM, Bezak E. Simulation of head and neck cancer oxygenation and doubling time in a 4D cellular model with angiogenesis. Scientific reports. 2017;7(1):1–11. Publisher: Nature Publishing Group.

14. Haustermans K, Vanuytsel L, Geboes K, Lerut T, Van Thillo J, Leysen J, et al. In vivo cell kinetic measurements in human oesophageal cancer: what can be learned from multiple biopsies? European Journal of Cancer. 1994;30(12):1787–1791. Publisher: Elsevier.

15. Choi SJ, Kim HS, Ahn SJ, Jeong YM, Choi HY. Evaluation of the growth pattern of carcinoma of colon and rectum by MDCT. Acta Radiologica. 2013;54(5):487–492. Publisher: SAGE Publications Sage UK: London, England.

16. Bolin S, Nilsson E, Sjödahl R. Carcinoma of the colon and rectum–growth rate. Annals of surgery. 1983;198(2):151. Publisher: Lippincott, Williams, and Wilkins.

17. Margolese R, HG BT. Natural history and prognostic markers. Holland-Frei Cancer Medicine 6th edition ed Hamilton (ON): BC Decker. 2003;.

18. Zharinov G, Gushchin V. The rate of tumor growth and cell loss in cervical cancer. Voprosy onkologii. 1989;35(1):21–25.

19. Carlson JA. Tumor doubling time of cutaneous melanoma and its metastasis. The American journal of dermatopathology. 2003;25(4):291–299. Publisher: LWW.

20. Harris K, Khachaturova I, Azab B, Maniatis T, Murukutla S, Chalhoub M, et al. Small cell lung cancer doubling time and its effect on clinical presentation: a concise review. Clinical Medicine Insights: Oncology. 2012;6:CMO–S9633. Publisher: SAGE Publications Sage UK: London, England.

21. Roesch K, Hasenclever D, Scholz M. Modelling lymphoma therapy and outcome. Bulletin of mathematical biology. 2014;76(2):401–430. Publisher: Springer.
